# Green Ammonia Generation with a Polypyridine Cobalt Complex and Visible Light

**DOI:** 10.1021/jacs.6c01477

**Published:** 2026-07-27

**Authors:** Andrea Mantovani, Federico Droghetti, Andreu Tortajada, Lubomír Rulíšek, Florian Lemken, Albert Ruggi, Mirco Natali

**Affiliations:** ‡ Department of Chemical, Pharmaceutical and Agricultural Sciences, 9299 University of Ferrara, Via L. Borsari 46, 44121 Ferrara, Italy; ⧫ 89220 Institute of Organic Chemistry and Biochemistry of the Czech Academy of Sciences, Flemingovo náměstí 2, 160 00 Praha 6, Czech Republic; ¶ Department of Chemistry, University of Fribourg, Chemin du Musée 9, 1700 Fribourg, Switzerland

## Abstract

The use of a first-row transition metal complex and visible light was exploited to promote the photosynthesis of ammonia from nitrite ions. This is achieved in neutral aqueous solution by employing a cobalt polypyridine complex (**CoL**) within a light-driven catalytic scheme involving [Ru­(bpy)_3_]^2+^ (where bpy = 2,2′-bipyridine) as the sensitizer and ascorbate as the electron donor. Under visible light irradiation, the resulting photosystem produces NH_3_ with a quantum yield up to 3% and a selectivity approaching 100%, achieving a maximum turnover number (TON) up to 2150. Transient absorption spectroscopy and density functional theory provided a robust mechanistic picture, showing that binding of the NO_2_^–^ substrate occurs at the one-electron reduced metal complex, while also ascertaining the energetic feasibility of subsequent elementary reaction steps. All in all, this work uncovers the great potential of first-row transition metal polypyridine complexes toward the synthesis of chemicals of industrial relevance.

## Graphical abstract

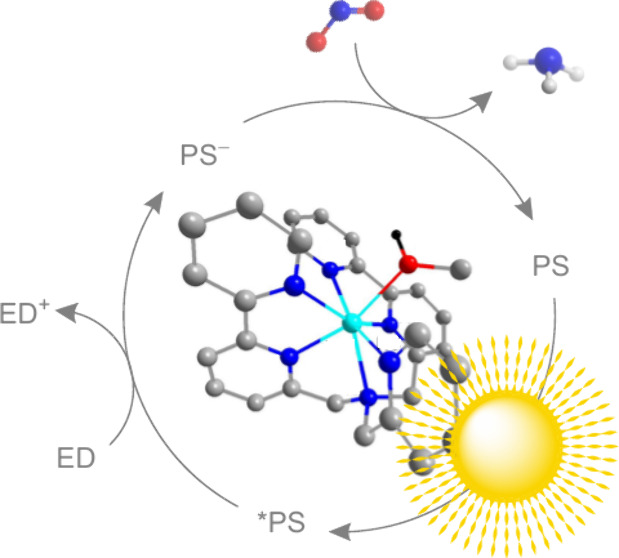

Ammonia (NH_3_) is one of the most important industrial chemicals worldwide, both in terms of production volume and breadth of applications.^1–3^ Industrial synthesis is, however, dominated by the Haber-Bosch process that still relies on fossil fuels.^2^ Decarbonizing NH_3_ synthesis has thus become a critical challenge.^4–6^ Recently, the nitrite reduction reaction (NO_2_^–^RR) has emerged as a viable alternative for the production of NH_3_.^7–10^ Cobalt complexes (Scheme 1a) have been employed as molecular catalysts to promote the NO_2_^–^RR from neutral aqueous solutions mainly resulting in the formation of NH_3_.^11–22^ However, the investigation of the activity has been solely limited to electrochemical conditions with the catalysts either used as homogeneous species in solution^11–18^ or immobilized onto solid-state supports.^19–22^ Notably, to the best of our knowledge, no transposition of this reactivity under light-driven conditions has been documented with only one reported molecular system employed for light-driven generation of NH_3_ based on a rare-earth element.^23^

**Scheme 1:**
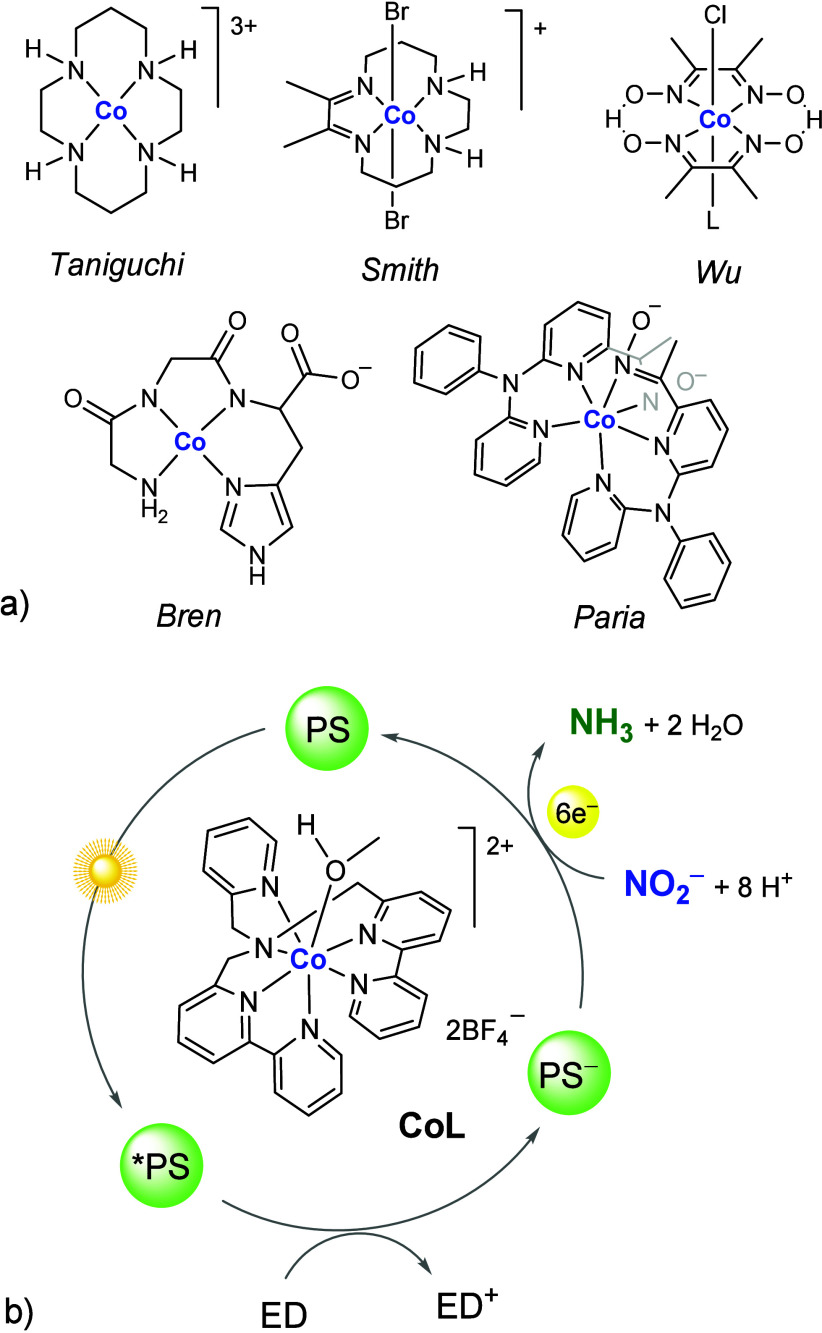
(a) Structures of Co Complexes Reported for Electrocatalytic NO_2_^–^RR and (b) Structure of **CoL** and Mechanism of Light-Driven NO_2_^–^RR to NH_3_ Presented in This Work^a^

a PS = Photosensitizer; ED = Electron Donor.

In recent years, we reported the use of metal complexes bearing the redox-active, hexadentate DBPy-PyA ligand (DBPy-PyA = (1-([2,2′-bipyridin]-6-yl)-*N*-([2,2′-bipyridin]-6-ylmethyl)-*N*-(pyridin-2-ylmethyl)­methanamine)) and related derivatives as catalysts for the hydrogen evolution reaction (HER)^24–27^ and CO_2_ reduction.^28–31^ Their great performance mainly stems from the redox-active nature of the ligand and its ability to dissociate under turnover conditions, creating internal proton relays for efficient proton transfer (PT) to the catalytic site, which is pivotal for achieving efficient catalysis.^32^ Herein, we show that the cobalt polypyridine complex (**CoL**) (Scheme 1b) can be a competent catalyst for selective reduction of NO_2_^–^ to NH_3_ in neutral aqueous solution. Its catalytic activity can also be transposed under light-driven conditions using [Ru­(bpy)_3_]^2+^ (where bpy = 2,2′-bipyridine) as the photosensitizer (PS) and ascorbate as the electron donor (ED), providing the first example of NH_3_ photosynthesis from aqueous NO_2_^–^ solution using a first-row transition metal complex.

**CoL** was obtained following a reported protocol, and the spectroscopic properties perfectly match the reported data.^24^ Cyclic voltammetry (CV) was performed in a phosphate buffer solution (0.25 M) at pH 7 (Figure 1a and SI for further details). Under these conditions, a hexacoordinate species, following removal of the monodentate ligand (i.e., a solvent molecule), is expected upon dissolution of **CoL**.^25,27^ DFT calculations indeed predict endergonic binding of a seventh ligand at the Co­(II) center.^31^ During the cathodic scan, a reduction process can be observed at −1.30 V vs SCE, which can be assigned, for comparison with the CV in acetonitrile,^31^ to a formal Co­(II)/Co­(I) electron transfer (ET) step. A current increase is then observed upon further cathodic scan without any apparent observation of clear reduction waves. This can be explained because, at more negative potentials than −1.40 V vs SCE, the expected reduction process, involving a formal Co­(I)/Co(0) step,^31^ is presumably coupled with PT to result in HER catalysis. This is indeed anticipated considering the reactivity of **CoL** in acetonitrile with weak organic acids.^33^ During the return scan, a sharp anodic peak is detected at −1.15 V vs SCE. Similar features were found in electrochemical studies in aqueous solutions for related cobalt complexes and attributed to stripping upon reoxidation of the neutral Co(0) complex adsorbed onto the electrode during the cathodic sweep.^34–36^ This feature disappears at more acidic pH (Figure S6), as Co­(I) protonation inhibits formation (and adsorption) of Co(0).^31,33^

**Figure 1:**
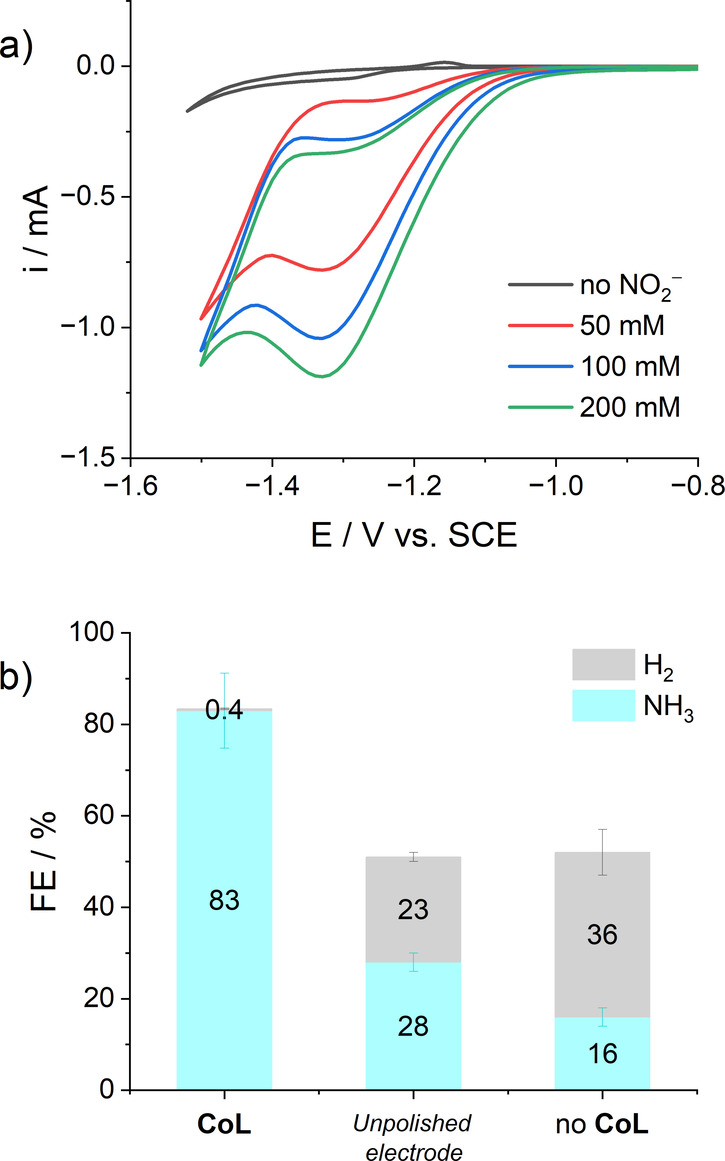
(a) CV of 1 mM **CoL** in 0.25 M phosphate buffer at pH 7 with 0–0.2 M NaNO_2_ at a scan rate of 0.1 V/s; (b) FEs of NH_3_ and H_2_ formation from CPE of 1 mM **CoL** in 0.25 M phosphate buffer at pH 7 with 0.05 M NaNO_2_ and control experiments (applied potential of −1.4 V vs SCE).

The electrochemical response of **CoL** was further assessed in the presence of nitrite to infer its catalytic ability toward the NO_2_^–^RR. Under these conditions, the comparison of the absorption spectra of **CoL** in 0.25 M phosphate buffer at pH 7 in the presence of NaNO_2_ (Figure S19) indicates that nitrite does not coordinate to Co­(II). Nevertheless, the addition of NO_2_^–^ triggers a current enhancement at the Co­(II)/Co­(I) reduction (Figure 1a), consistent with nitrite binding at the one-electron reduced species and ensuing catalysis. The current measured slightly increases with the concentration of the substrate leading to an *i*_c_/*i*_p_ ratio up to 17.6 with 0.2 M NaNO_2_. At fixed NO_2_^–^ content, the current increases with decreasing pH and increasing phosphate buffer concentration (Figures S9 and S10), supporting the key role of proton delivery in the catalytic process.^13,14,37^ Control tests (Figure S11) finally confirm that **CoL** is necessary to trigger any current enhancement.

Controlled-potential electrolysis (CPE) was performed to determine and quantify the reduction products. The results (Figure 1b) show that NH_3_ is formed as virtually the sole reduction product with a faradaic efficiency (FE) of 83%. No NH_2_OH is detected, while H_2_ is generated with an FE of only 0.4%. Importantly, when CPE was conducted in the absence of **CoL**, the FE for NH_3_ formation drastically decreased to only 16%, whereas the FE for H_2_ increased to 36% (Figure 1b). Furthermore, an FE of 28% for NH_3_ and a parallel FE of 23% for H_2_ were measured during electrolysis of a catalyst-free solution using the electrode previously employed in the CPE with **CoL** (unpolished electrode). All of these controls confirm that selective NH_3_ production requires the presence of **CoL** and does not originate from heterogeneous species formed on the electrode. Notably, the large FE for NH_3_ production is in good agreement with the constant current response (Figure S12), supporting the stability of **CoL** under operation.

We then examined the possibility of extending this reactivity under light-driven conditions. To this purpose, we combined **CoL** with [Ru­(bpy)_3_]^2+^ as the sensitizer and ascorbate as the electron donor.^38^ We first started by evaluating the effect of the concentration of **CoL** under 460 nm irradiation of 0.25 M phosphate buffer solutions at pH 7 containing 0.5 mM [Ru­(bpy)_3_]^2+^, 0.1 M ascorbate, and 0.05 M NaNO_2_. Figure 2a collects the results in terms of amount of NH_3_ produced after 23 h of irradiation within a concentration range spanning from 2 μM up to 100 μM **CoL**. The activity in terms of product formation peaks at intermediate concentrations (Figure 2a), while the turnover number (TON) maximizes at 2 μM **CoL**, achieving a remarkable value of 2150 (Figure S15). We next selected a concentration of 25 μM **CoL** for further assays. Figure 2b depicts the kinetics of NH_3_ formation as a function of the irradiation time. Production of NH_3_ is appreciably linear in time within the first 10 h, and a quantum yield of 3% can be estimated (see SI). NH_3_ photogeneration then starts slowing down at longer irradiation times and finally reaches a plateau producing up to 43 μmol of NH_3_ after 72 h. NH_3_ is produced with nearly 100% selectivity, as no NH_2_OH is detected via ^1^H NMR and only 0.1 μmol of H_2_ is measured by GC after 23 h.

**Figure 2:**
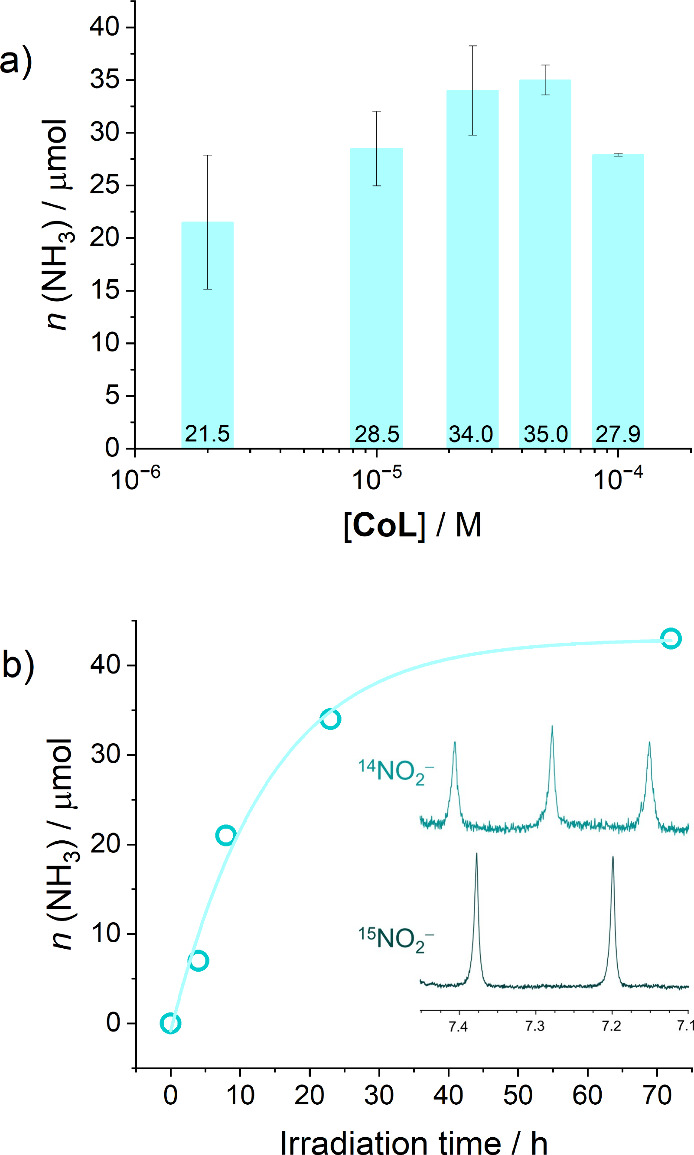
(a) Amount of NH_3_ generated after 23 h of irradiation (LED @460 nm) of 0.25 M phosphate buffer solution (5 mL) at pH 7 containing 0.5 mM [Ru­(bpy)_3_]­Cl_2_·6H_2_O, 0.1 M sodium ascorbate, 0.05 M NaNO_2_, and 2–100 μM **CoL**; (b) kinetics of NH_3_ formation at [**CoL**] = 25 μM and comparison of the ^1^H NMR signal associated with NH_4_^+^ measured with 0.05 M Na^14^NO_2_ or Na^15^NO_2_ (inset).

Notably, addition of fresh [Ru­(bpy)_3_]^2+^ after 23 h of irradiation fully restores the activity, leading to a cumulative NH_3_ production of 72 μmol after 46 h of irradiation (Figure S16). These findings confirm catalyst stability and identify degradation of the sensitizer as the main limiting factor.^39^ Comparison of the absorption spectra before and after photocatalysis shows a marked attenuation of the MLCT transition of the chromophore (Figure S17), thus corroborating this interpretation.

NH_3_ photogeneration after 23 h of irradiation is maximized at pH 7 (Table S1) and, at this pH, is only weakly dependent on both NO_2_^–^ and phosphate buffer concentrations (Tables S2 and S3). The similar performance recorded at different nitrite loadings is consistent with the modest NO_2_^–^ conversion recorded at the plateau (17%), whose value points out how substrate consumption does not represent a limiting factor herein. Control experiments (Table S4) confirm that **CoL**, [Ru­(bpy)_3_]^2+^, and ascorbate are all simultaneously required for the effective generation of NH_3_. Furthermore, experiments conducted with Na^15^NO_2_ show the formation of isotopically labeled ^15^NH_3_ (Figure 2b, inset), thus demonstrating that it unequivocally originates from the reduction of nitrite.^22^ As a final remark, the NH_3_ photoproduct can also be quantitatively separated in the form of NH_4_Cl salt, devoid of impurities from the photolyzed solution (Figure S18), upon vacuum transfer of the photolyzed mixture into a 3 M HCl aqueous solution, highlighting how the homogeneous approach used herein can also lead to clean and quantitative product capture.

We next performed spectroscopic studies to attain mechanistic insights. Figure 3a depicts the laser flash photolysis (LFP) experiments conducted on a 0.25 M phosphate buffer solution at pH 7 with [Ru­(bpy)_3_]^2+^, ascorbate, and **CoL**.

**Figure 3:**
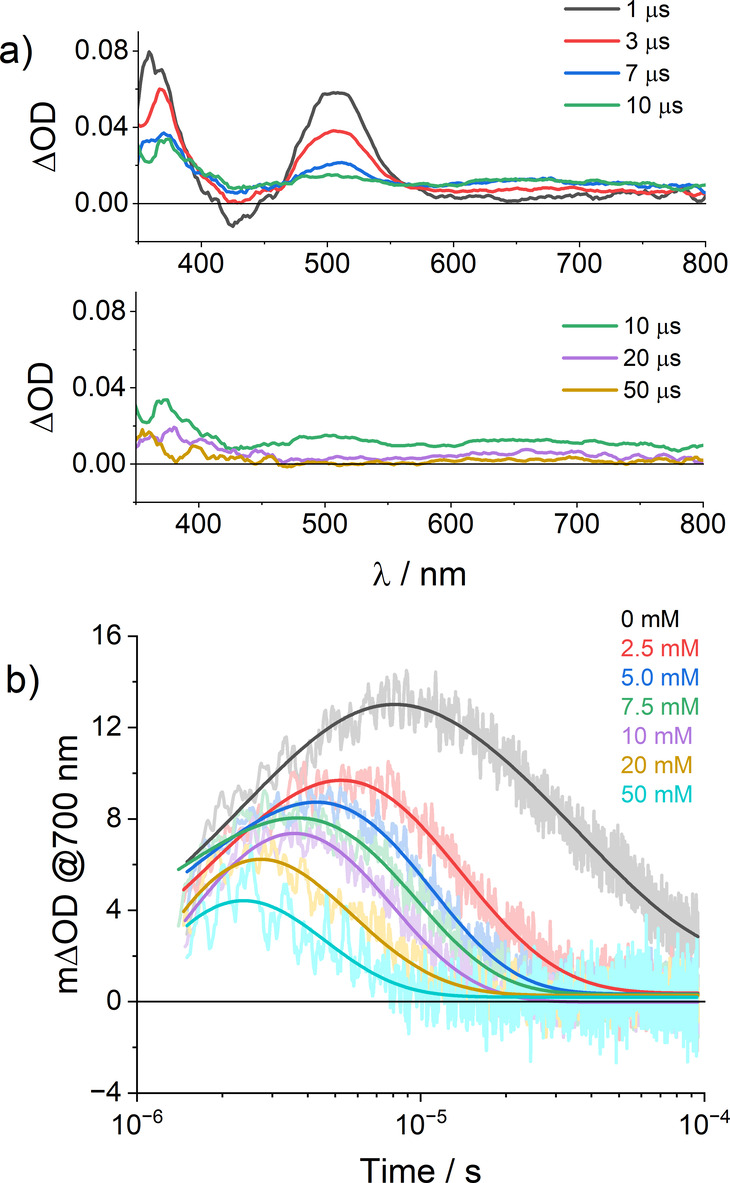
(a) Transient absorption spectra between 1 and 50 μs measured by LFP (excitation at 532 nm) of an N_2_-purged 0.25 M phosphate buffer solution at pH 7 containing 70 μM [Ru­(bpy)_3_]­Cl_2_·6H_2_O, 0.1 M sodium ascorbate, and 100 μM **CoL** and (b) kinetic traces at 700 nm with 0–50 mM NaNO_2_.

The transient spectrum measured at 1 μs (Figure 3a, top) features an absorption band at 500 nm which corresponds to the reduced [Ru­(bpy)_3_]^+^ species^39,40^ obtained by reductive quenching of the excited sensitizer with the ascorbate donor.^41,42^ This absorption progressively decreases in the following 10 μs (Figure 3a, top), leading to the concomitant formation of a broad transient absorption characteristic of the reduced **CoL**,^25,27,43–45^ which is identified as a triplet ground state via the Evans method (Figure S24), in agreement with the calculations (Tables S5–S7).^31,33^ This residual absorption finally decays to the baseline within 50 μs (Figure 3a, bottom) mainly due to recombination between Co­(I) and the ascorbate radical.^33,43^ Interestingly, the transient absorption signal at 700 nm, associated with the reduced **CoL**, undergoes a faster decay in the presence of NaNO_2_ (Figure 3b). This evidence can be explained by assuming binding of NO_2_^–^ to the formal Co­(I) generated upon ET from [Ru­(bpy)_3_]^+^. UV–vis spectroelectrochemistry (SEC) in acetonitrile confirms the abatement of the absorption characteristic of reduced **CoL** in the presence of 0.05 M TBANO_2_ (Figure S22), supporting the proposed mechanistic hypothesis. Interestingly, the rate of this reaction increases with NO_2_^–^ concentrations for values below 0.01 M, finally reaching a plateau above 0.05 M. Fitting of the observed rate *k* as a function of [NO_2_^–^] was attempted using an empirical saturation model (Figure S23), which allows one to extract an apparent maximum velocity of 3.5·10^5^ s^–1^ for nitrite coordination at the reduced **CoL** under conditions relevant to the photocatalytic assays. The apparent saturation attained at larger NO_2_^–^ concentrations corroborates the experimental observation of a light-driven catalytic activity independent of substrate loading in the range of 0.05–0.5 M (Table S3).

DFT-D3//COSMO-RS calculations (see SI for computational details) were finally conducted to propose a catalytic mechanism. A schematic representation, with relevant structures and free energy changes of key chemical steps, is presented in Scheme 2, while details of structural and energetic parameters are provided in Scheme S1 and Figure S26. As confirmed by LFP, nitrite binding occurs at Co­(I) (+12.4 kcal·mol^–1^). The complex then evolves through several intermediates, whose formation, implying PT and dehydration steps, is generally thermodynamically favorable (see SI). All these processes ultimately lead to a Co-amino species from which NH_3_ release can occur. This step is exergonic by −8.0 kcal·mol^–1^, supporting efficient product release. In contrast, computations predict an endergonic NH_2_OH release (+8.3 kcal·mol^–1^), mainly ascribed to favorable H-bonding interactions with a dangling pyridine, corroborating the selective formation of NH_3_, as experimentally observed.

**Scheme 2:**
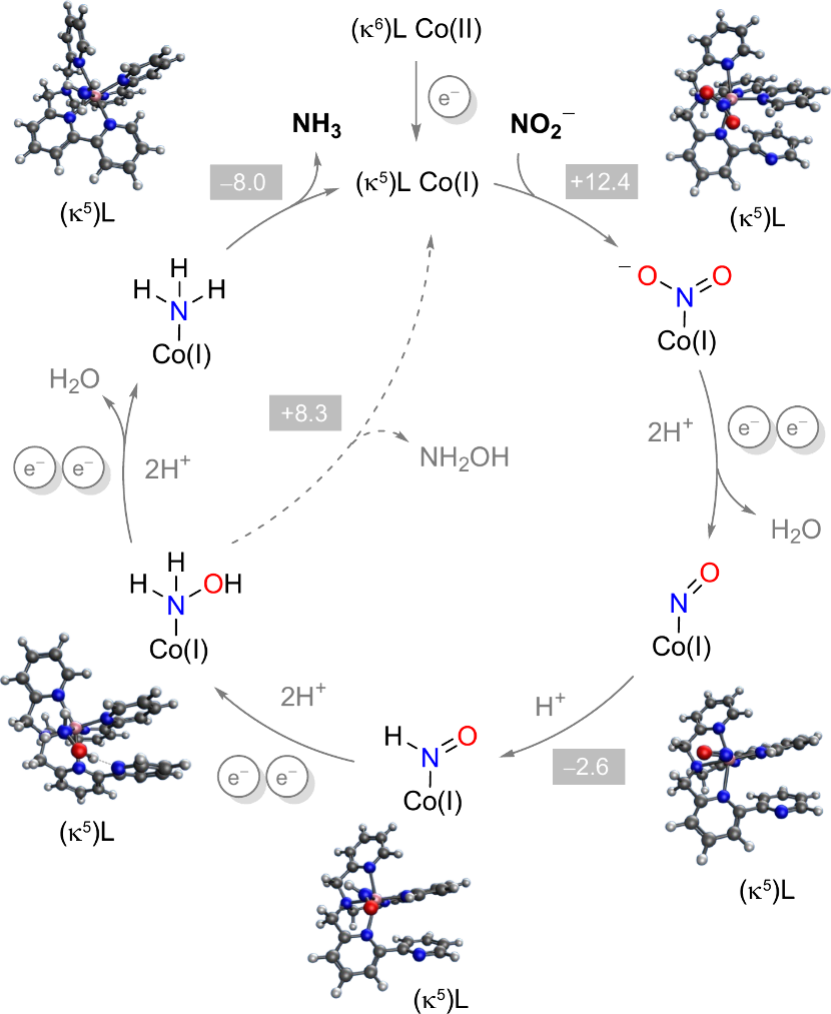
Proposed Catalytic Mechanism for NO_2_^–^RR to NH_3_ by **CoL** with Structures of Relevant Intermediates and Free Energy Changes (in kcal·mol^–1^) Associated with Key Chemical Steps^a^

a See SI for details.

In conclusion, we reported for the first time the integration of molecular catalysis by a first-row transition metal complex and visible light to promote selective conversion of NO_2_^–^ into NH_3_. This is achieved in aqueous solution at neutral pH by combining the cobalt complex **CoL** with [Ru­(bpy)_3_]^2+^ as the photosensitizer and ascorbate as the electron donor. This work underscores the great potential of polypyridine complexes for advancing sustainable synthetic methods relevant to high-value industrial products.

## Supplementary Material





## Notes

Experimental and computational methodologies, additional electrochemical measurements, additional photochemical studies, additional mechanistic data, and detailed catalytic mechanism (PDF)
Computational data: coordinates of all stationary points and their respective energies (ZIP)

## Abbreviations

NO_2_^–^RR: nitrite reduction reaction
HER: hydrogen evolution reaction
PT: proton transfer
ET: electron transfer
PS: photosensitizer
ED: electron donor
CV: cyclic voltammetry
CPE: controlled potential electrolysis
LFP: laser flash photolysis
GC: gas chromatography
SEC: spectroelectrochemistry
