# Supplementary material for: Green Ammonia Generation with a Polypyridine Cobalt Complex and Visible Light
Source: J Am Chem Soc. 2026 Jul 27;148(30):31621–6. doi: 10.1021/jacs.6c01477 (PMC13449952; doi:10.1021/jacs.6c01477)
Supplement: Supplementary file 1 [file ja6c01477_si_001.pdf]

Supporting Information

Green Ammonia Generation with a Polypyridine Cobalt Complex and Visible Light

Andrea Mantovani,[‡] Federico Droghetti,[‡] Andreu Tortajada,[¶] Lubomír Rulíšek,[♦] Florian Lemken,[♦] Albert Ruggi,[¶] and Mirco Natali[‡]**

[‡] Department of Chemical, Pharmaceutical and Agricultural Sciences University of Ferrara Via L. Borsari 46, 44121 Ferrara, Italy.

E-mail: mirco.natali@unife.it

[♦] Institute of Organic Chemistry and Biochemistry of the Czech Academy of Sciences, Flemingovo náměstí 2, 160 00, Praha 6, Czech Republic.

[¶] Department of Chemistry, University of Fribourg, Chemin du Musée 9, 1700 Fribourg, Switzerland.

E-mail: albert.ruggi@unifr.ch

Table of Contents

S1. Materials and methods p. S2

S2. Electrochemistry p. S8

S3. Light-driven catalysis p. S15

S4. Mechanistic investigation p. S21

S5. Computational studies p. S27

S6. References of the Supporting Information p. S32

S1. Materials and methods

S1.1 Apparatuses and procedures

All reagents were obtained from standard suppliers and used without additional purification. **CoL** was prepared according to literature procedures.[S1]

Absorption spectra were recorded at room temperature using an Agilent Technologies spectrophotometer.

Transient absorption spectroscopy experiments were conducted using a custom laser spectrometer comprising a Continuum Surelite II Nd: YAG laser (FWHM = 8 ns) with a frequency-doubled option (532 nm). Photomultiplier signals (kinetic traces) were processed by using a Teledyne LeCroy 604Zi (400 MHz, 20 GS/s) digital oscilloscope. Transient spectra were recorded using a PiMax CCD Camera.

^{1}H-NMR spectra were registered on a Varian Mercury Plus 400 spectrometer. Chemical shifts are given in ppm relative to the NMR standard tetramethylsilane (TMS), and J values are given in Hz. Estimation of the number of unpaired electrons in both **CoL** and its reduced form was performed with the Evans method. Solutions were prepared in an Ar glove box (MBraun) by using d^6-DMSO distilled over CaH_2 and stored over dry molecular sieves. Evans method experiments were performed in J Young NMR tubes (purchased from GPE Scientific) to prevent contact with air, using a Bruker Avance III spectrometer operating at 500 MHz. Mg-anthracene was used as chemical reducing agent which was synthesized and characterized according to the literature.[S2] For the calculation of the magnetic moment and estimation of the number of unpaired electrons before and after reduction, a tube containing 9.8 mg (0.014 mmol) of **CoL** in 0.5 mL d^6-DMSO was prepared in a glove box. A flame-sealed coaxial tube containing d^6-DMSO and 5% of non-deuterated DMSO was added to the tube to use the chemical shift of DMSO as reference value for the solvent in non-paramagnetic environment. After measurement with **CoL**, the tube was loaded with 7.4 mg (0.018 mmol; 1.3 eq.) of Mg-anthracene and its ^{1}H-NMR spectrum was recorded again.

Cyclic voltammetry measurements were conducted on a PGSTAT302N potentiostat (Autolab) in a three-electrode configuration, with glassy carbon as a working electrode, SCE as a reference electrode, and Pt as a counter electrode. The solution was purged for 20 minutes with Ar before starting the measurement.

Spectroelectrochemistry was performed using a commercial OTTLE cell (ProSense) using a Pt grid as the working electrode, a silver wire as the quasi-reference electrode and a Pt wire as counter electrode. The applied potential was set at the Co(II)/Co(I) reduction by recording a CV experiment prior to electrolysis.

Controlled potential electrolysis (CPE) experiments were performed in a gas-tight custom-made electrochemical cell using a high surface area glassy-carbon rod as a working electrode, a Pt electrode as a counter, and a saturated calomel electrode (SCE) as a reference. The CPE experiments for the **CoL** sample, the unpolished electrode, and the blank were run by allowing the same final charge to be passed. The results are the average of two independent experiments.

Light-driven catalytic experiments were performed under monochromatic irradiation (LED at 460 nm, 3 W) of a 5 mL solution containing the proper amounts of **CoL**, $[Ru(bpy)_3]Cl_2 \cdot 6H_2O$, sodium ascorbate, and $NaNO_2$. The solutions were purged with Ar for 20 minutes before irradiation. A cylindrical cell was employed, and irradiation was provided from the bottom. All the photocatalytic experiments were run in duplicate, and the results are the average of two independent experiments.

S1.2 Computational methods

All geometry optimizations and electronic structure calculations used the Turbomole-7.7 program package.[S3] Geometry optimizations were performed at the BP86-D3(BJ)/def2-TZVP level of theory[S4-S12]. The minimum energy geometries of all intermediates were chosen from an ensemble of trial conformers, containing both the heptacoordinated and several hexacoordinated versions of the intermediates. Electronic structure calculations were performed at the B3LYP-D3(BJ)/def2-TZVPD

level of theory.[S4-S13] The spin multiplicity of the ground state of each intermediate was determined at the B3LYP*-D3(BJ)/def2-TZVPD level of theory.[S14-S16] This functional has been optimized for the calculation of spin crossover energies, and was found[S17,S18] to well reproduce CASPT2+δMRCI calculations of such energies for cobalt complexes. Vibrational frequencies were calculated with Turbomole-7.7 and converted into vibration free energies with the thermo submodule of the XTB software package.[S13] Therein, an empirical scaling factor of 0.9914 was applied to calculated frequencies. Solvation free energies were computed using the COSMO-RS method,[S20,S21] using the COSMOtherm23 software package.[S22] The solvation free energy of a proton in water under standard conditions is difficult to determine with standard quantum chemical methods.[S23] Thus, an experimental estimate of $\Delta_S G(H^+) = -265.9$ kcal·mol^{-1} based on the cluster pair approximation was used.[S24] For consistency with experimental reduction potentials, calculated reduction potentials are displayed relative to the SCE reference of 4.50 V.[S25] Finally, a correction of 1.9Δn kcal·mol^{-1} (corresponding to the difference between the concentration of the ideal gas at 298 K and 1 atm and its 1 mol·L^{-1} concentration; Δn is the change in the number of moles in the reaction)[S24-S26] has been applied so that the computed values refer to the 1 mol·L^{-1} standard state.

S1.3 Product detection and quantification

S1.3.1 NH_3 detection

NH_3 was detected in the form of NH_4^+ ion by ^{1}H-NMR and quantified using maleic acid as an internal standard (δ = 6.2 ppm, s, 2H). 68 µL of the sample solution was acidified with 68 µL of a solution containing 2 M HCl and 20 mM maleic acid. The mixture was diluted with 750 µL of DMSO-d_6, and the resulting solution was analyzed by ^{1}H-NMR (400 MHz). NH_4^+ was observed at δ = 7.1-7.5 ppm (t, 4H, J = 53 Hz) in agreement with a control NMR experiment performed on a NH_4Cl aqueous solution (Figure S1).[S27]

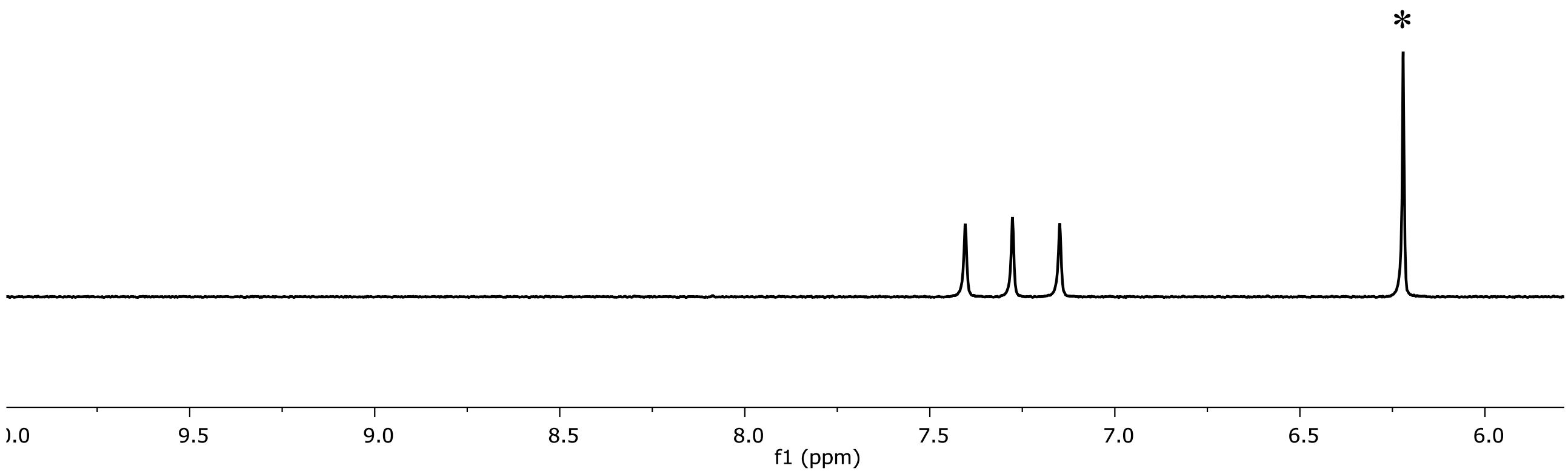

Figure S1. ^{1}H-NMR spectrum in DMSO-d_6 of an aqueous solution containing 14 mM NH_4Cl together with the maleic acid standard (*).

Detection and quantification of NH_4^+ was also confirmed by colorimetric assay using the indophenol blue method.[S28] Briefly, 20 µL of the sample solution were added to 5 mL of solution A (containing 0.1 M phenol and 0.167 mM sodium nitroprusside). Subsequently, 5 mL of solution B (an alkaline solution composed of 0.125 M NaOH and 0.124 M NaClO) were added, and the resulting mixture was stirred for 1 h in the dark at room temperature. After incubation, the absorbance maximum was recorded at 630 nm. Quantification was performed via calibration using aqueous NH_4Cl solutions (Figure S2). The resulting calibration curve follows an appreciable linear trend within a concentration range from 0 to 70 µM (Figure S3). Gratifyingly, the average error between the two quantification methods was found in the order of ca 3%.

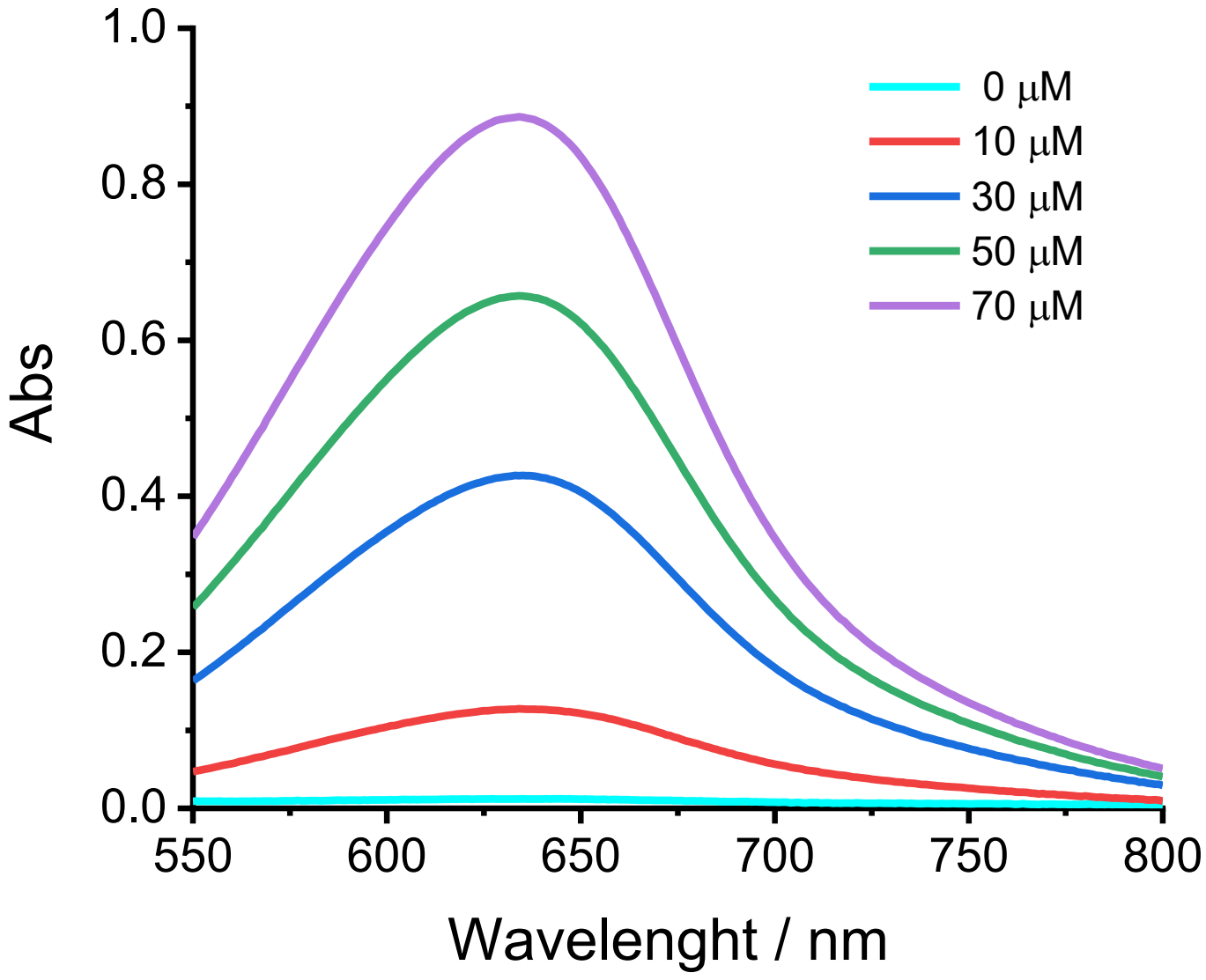

Figure S2. Absorption spectra of aqueous solutions containing 0-70 μM NH_4Cl treated according to the indophenol method.

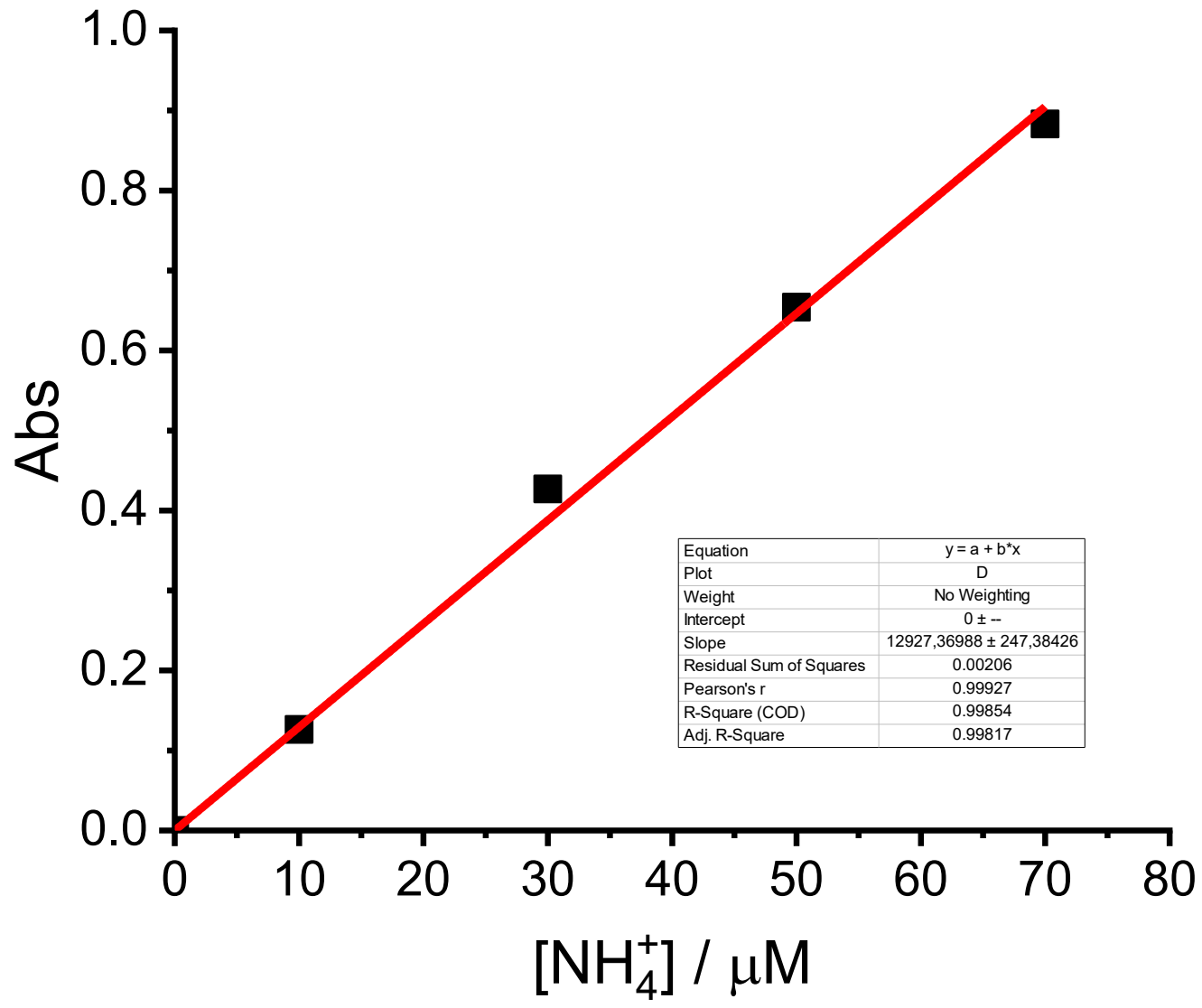

Figure S3. Calibration curve, obtained by plotting the absorbance at 630 nm as a function of NH_4Cl concentration, used to confirm the amount of NH_4^+ formed in the electrochemical and photochemical experiments.

S1.3.2 NH_2OH detection

NH_2OH was detected in the form of NH_3OH^+ ion by ^{1}H-NMR and quantified using maleic acid as an internal standard (δ = 6.2 ppm, s, 2H). 68 μL of the sample solution was acidified with 68 μL of a solution containing 2 M HCl and 20 mM maleic acid. The mixture was diluted with 750 μL of DMSO-d^6, and the resulting solution was analyzed by ^{1}H-NMR (400 MHz).

NH_3OH^+ was observed at δ = 10.5 ppm (3H, s) and δ = 10.1 ppm (H, s), according to the NMR measurement on a NH_3OHCl aqueous solution (Figure S4).

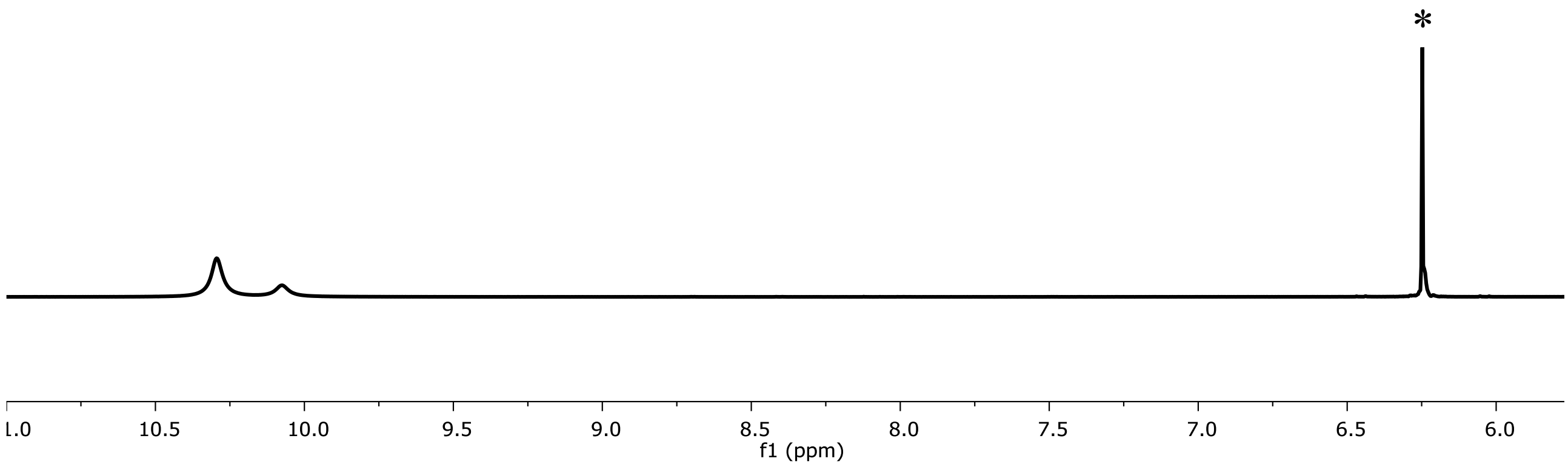

Figure S4. ^{1}H-NMR spectrum in DMSO-d_6 of an aqueous solution containing 10 mM NH_3OHCl together with the maleic acid standard (*).

S1.3.3 H_2 detection

H_2 was detected using an Agilent Technologies 490 micro-GC, equipped with a 5 A molecular sieve column (10 m), a thermal conductivity detector, and using Ar as the carrier gas. The moles of H_2 were quantified through the external calibration method through a galvanostatic (typically 1 mA) electrolysis of a 0.1 M H_2SO_4 solution. A 100% Faradaic efficiency was assumed leading to a linear correlation between the amount of H_2 evolved at the cathode and the electrolysis time.

S2. Electrochemical characterization

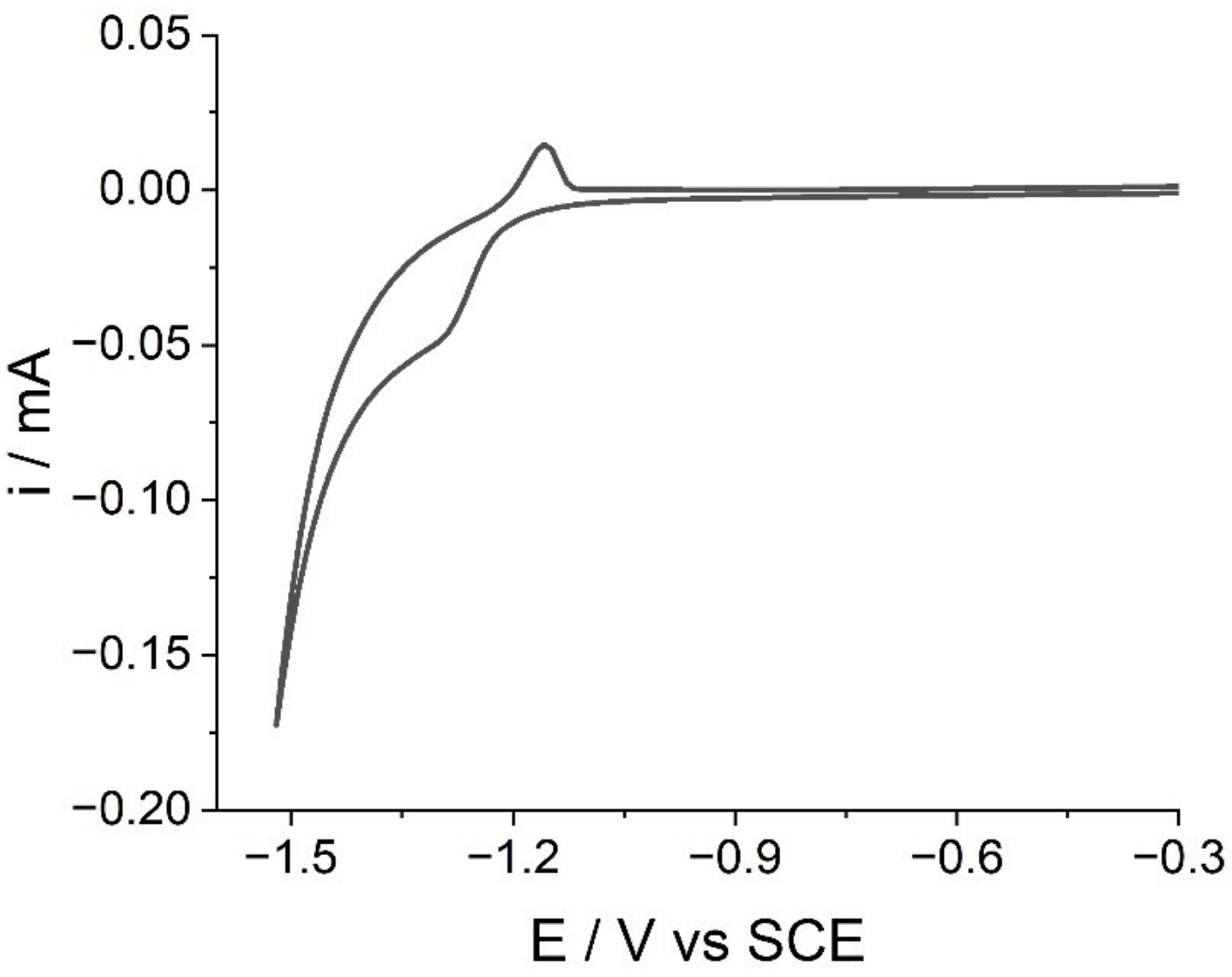

Figure S5. CV of 1 mM **CoL** in 0.25 M phosphate buffer solution at pH 7 at a scan rate of 0.1 V/s.

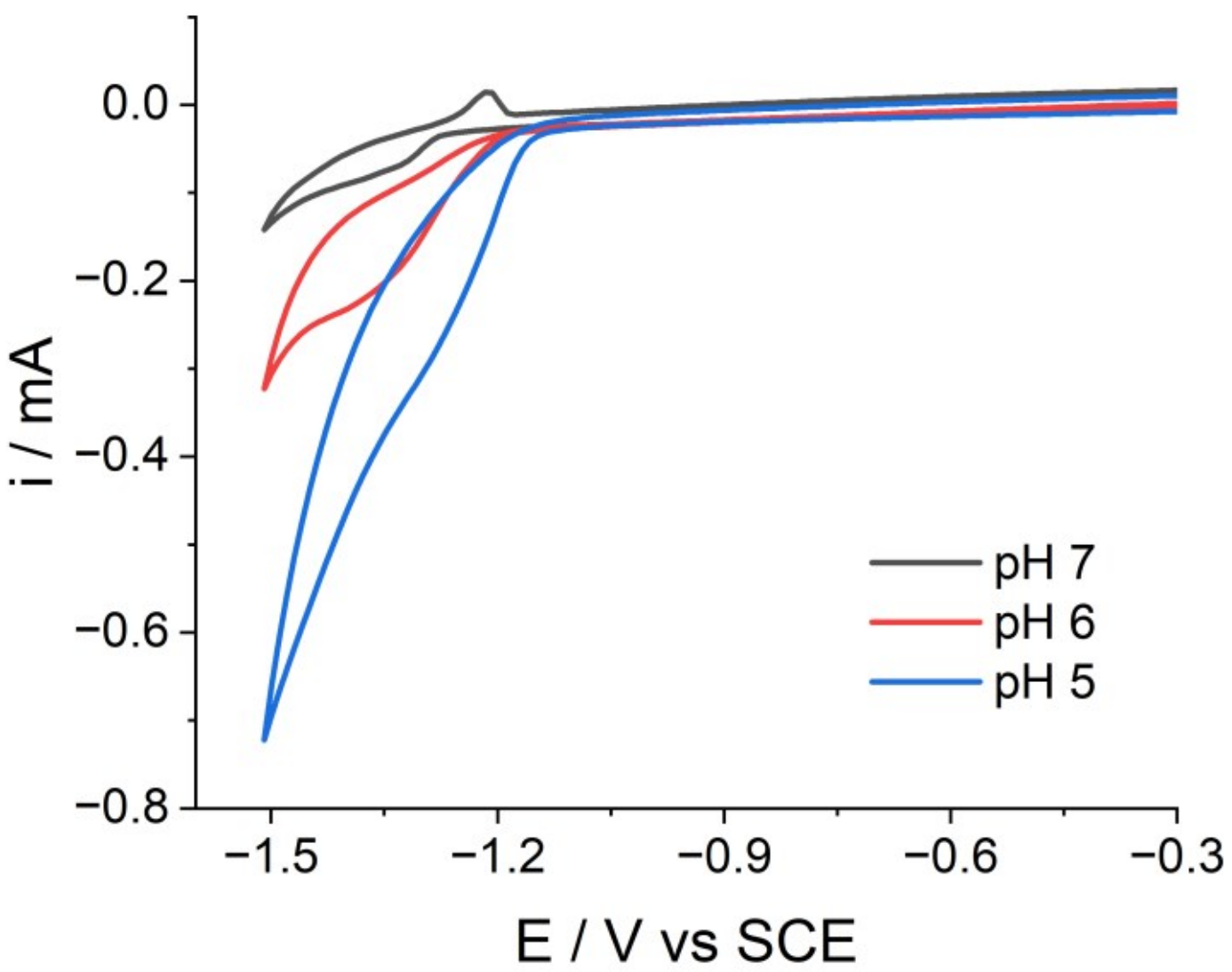

Figure S6. CVs of 1 mM **CoL** in 0.25 M phosphate buffer solution at a scan rate of 0.1 V/s at different pH values.

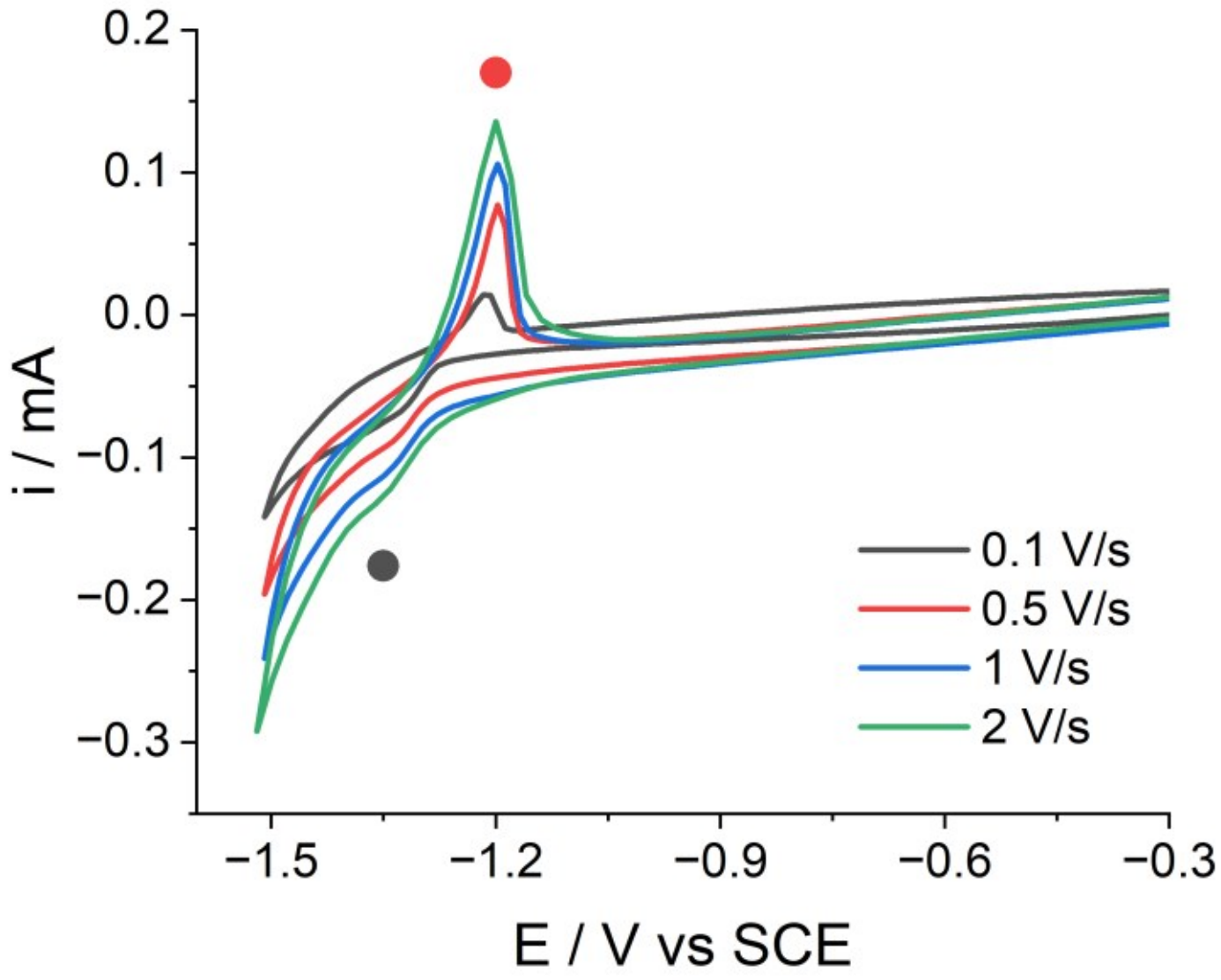

a)

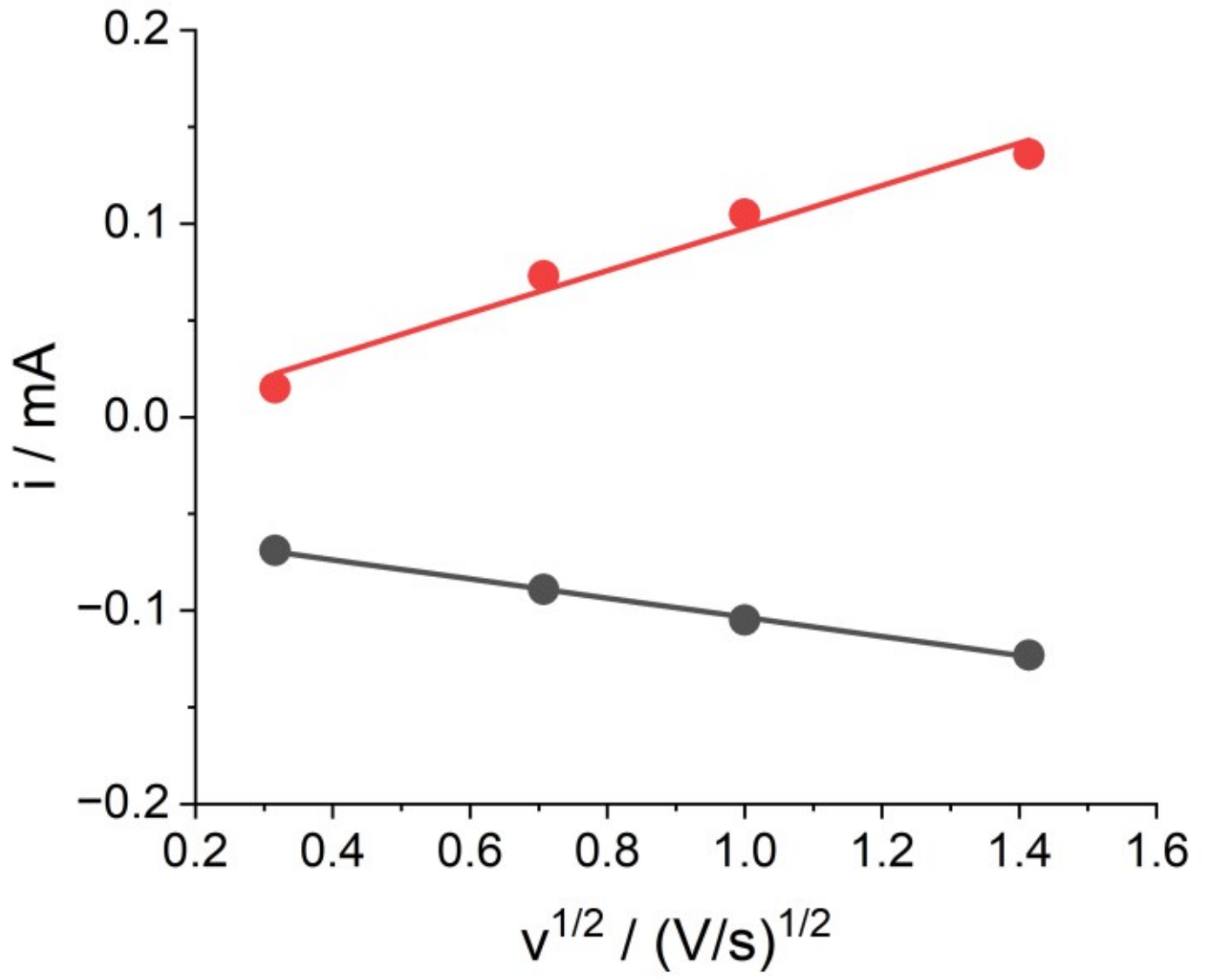

b)

Figure S7. a) CVs of 1 mM **CoL** in 0.25 M phosphate buffer solution at pH 7 at variable scan rates and b) plot of the cathodic (black circles) and anodic (red circles) peak currents as a function of the square root of the scan rate.

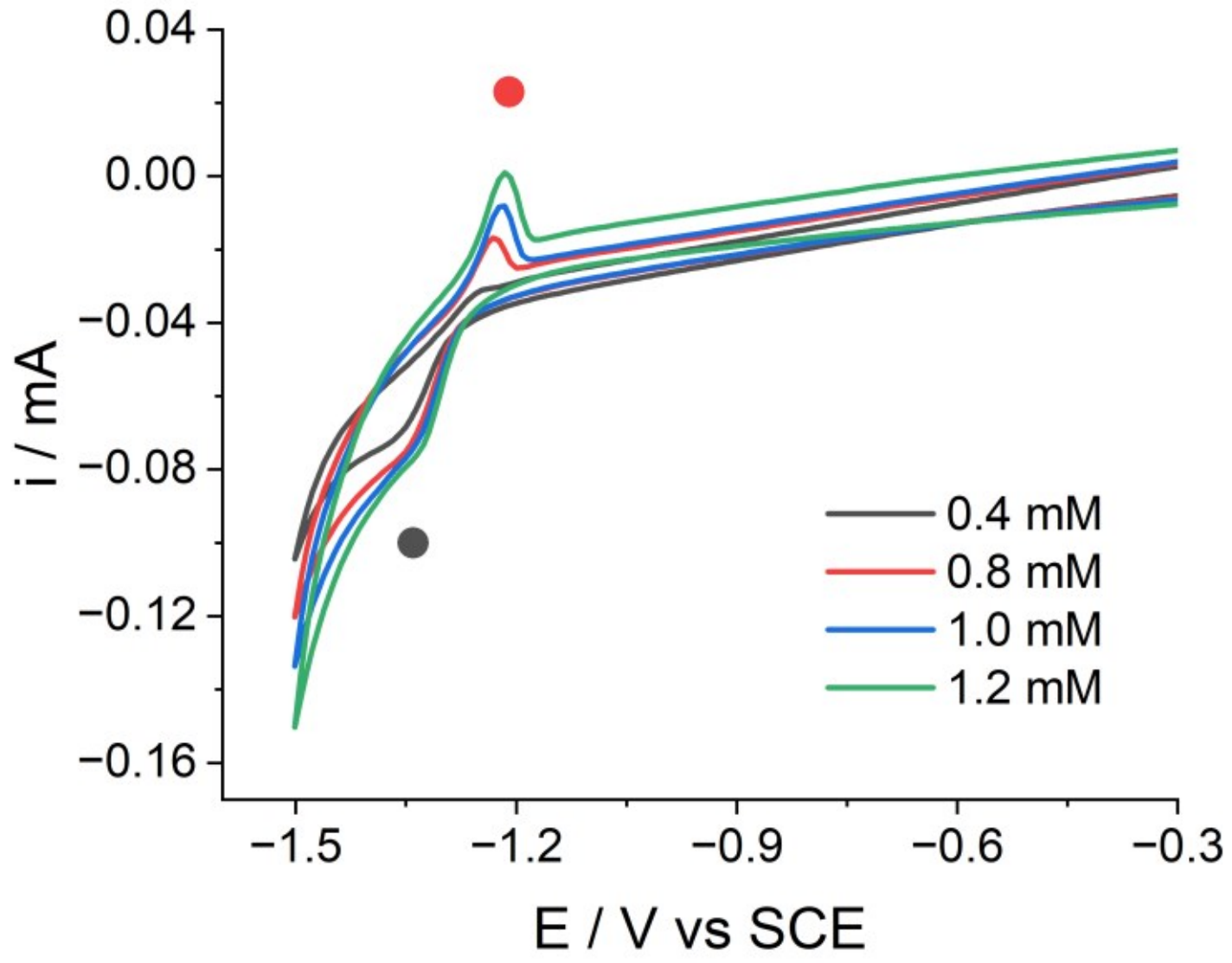

a)

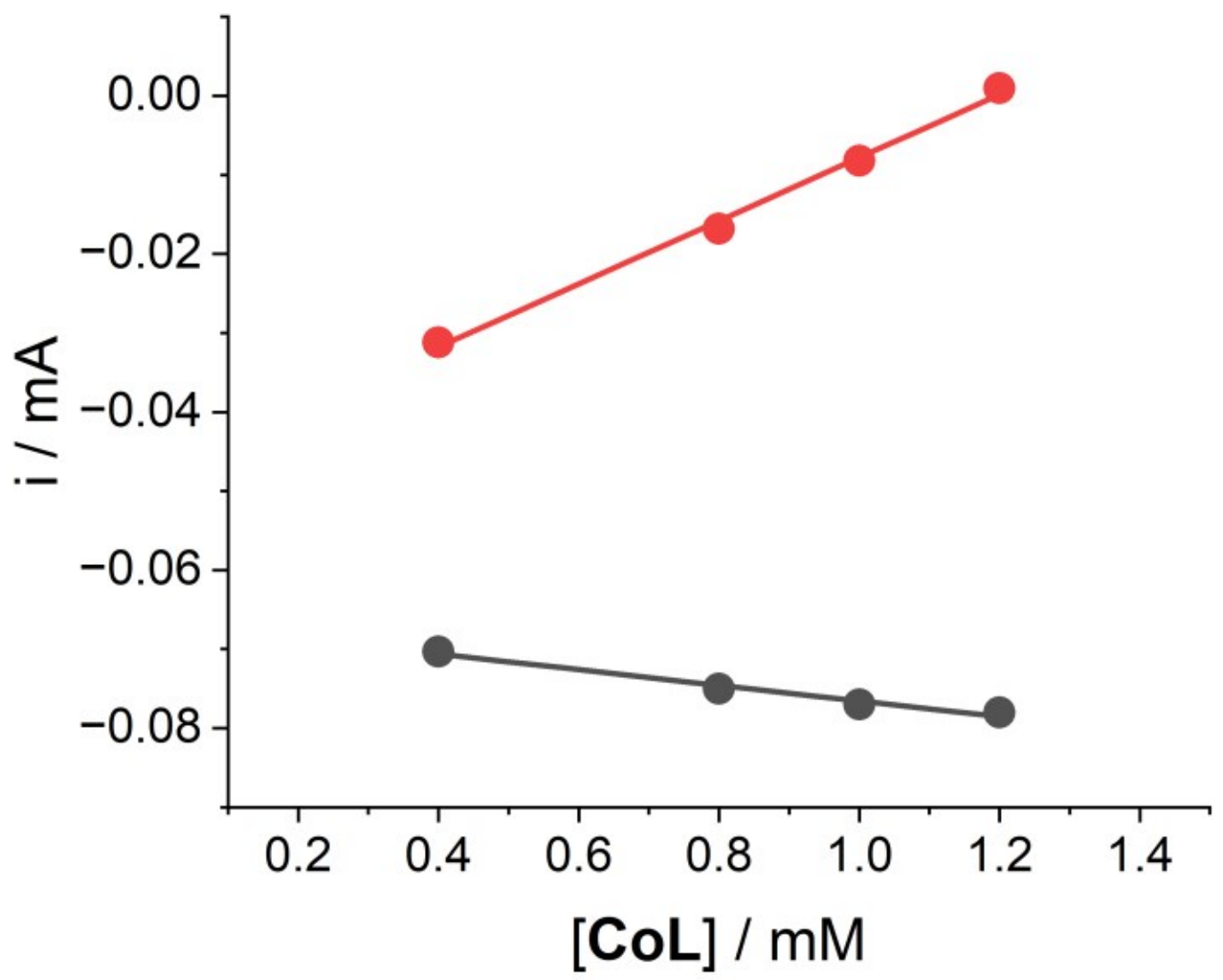

b)

Figure S8. a) CVs of **CoL** in 0.25 M phosphate buffer solution at pH 7 at a scan rate of 0.1 V/s at variable concentrations and b) plot of the cathodic (black circles) and anodic (red circles) peak currents as a function of the square root of the scan rate. The shape of the anodic current is consistent with stripping of adsorbed species from the electrode assigned to neutral Co(0) reduced species.[S29-S31] The linear dependence of the peak current on both the square root of the scan rate (Figure S7) and **CoL** concentration (Figure S8) is indicative of a fast, reversible process.

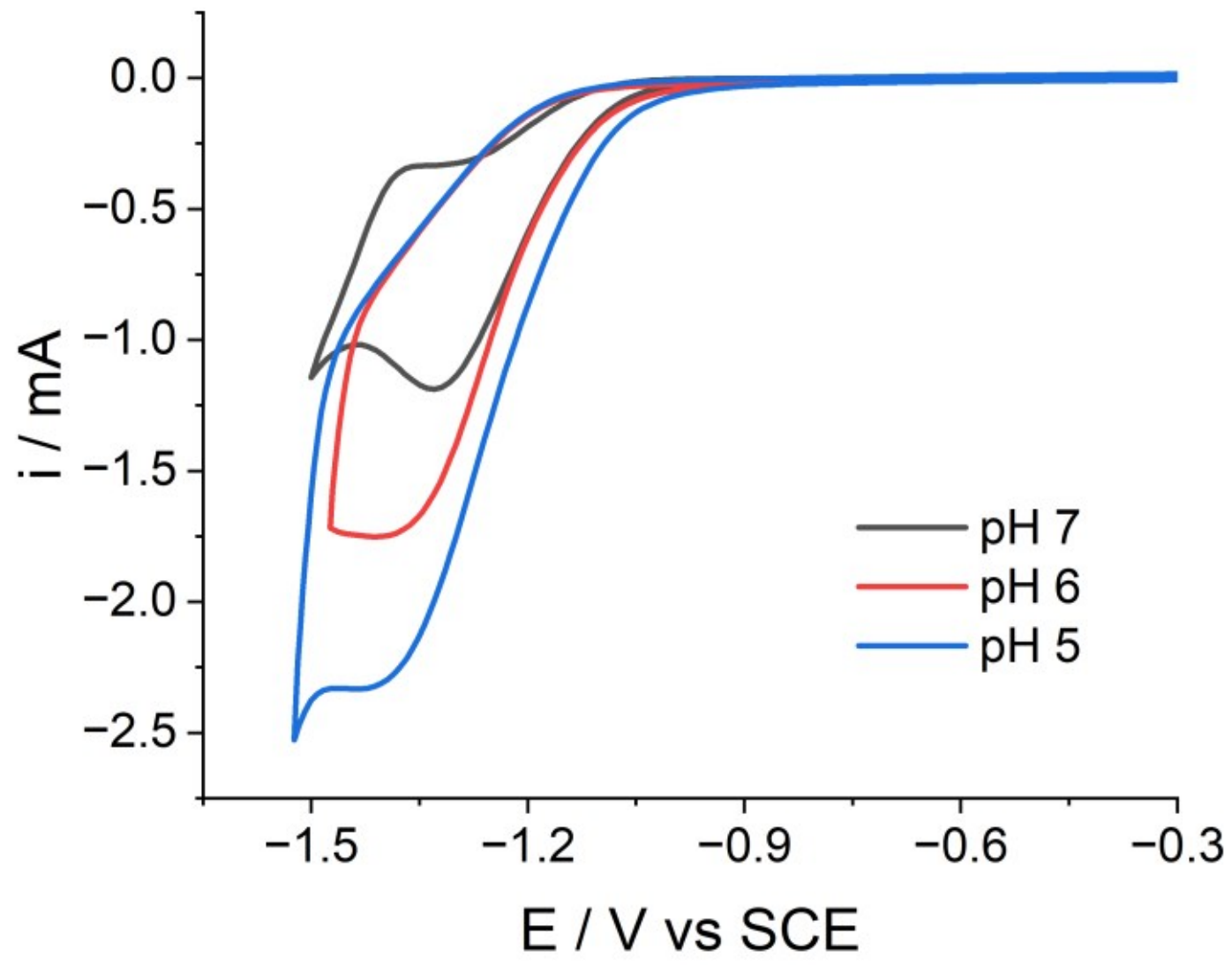

Figure S9. Comparison of CVs experiments recorded in 0.25 M phosphate buffer solution at a scan rate of 0.1 V/s in the presence of 200 mM $NaNO_2$ and 1 mM **CoL** at different pH values.

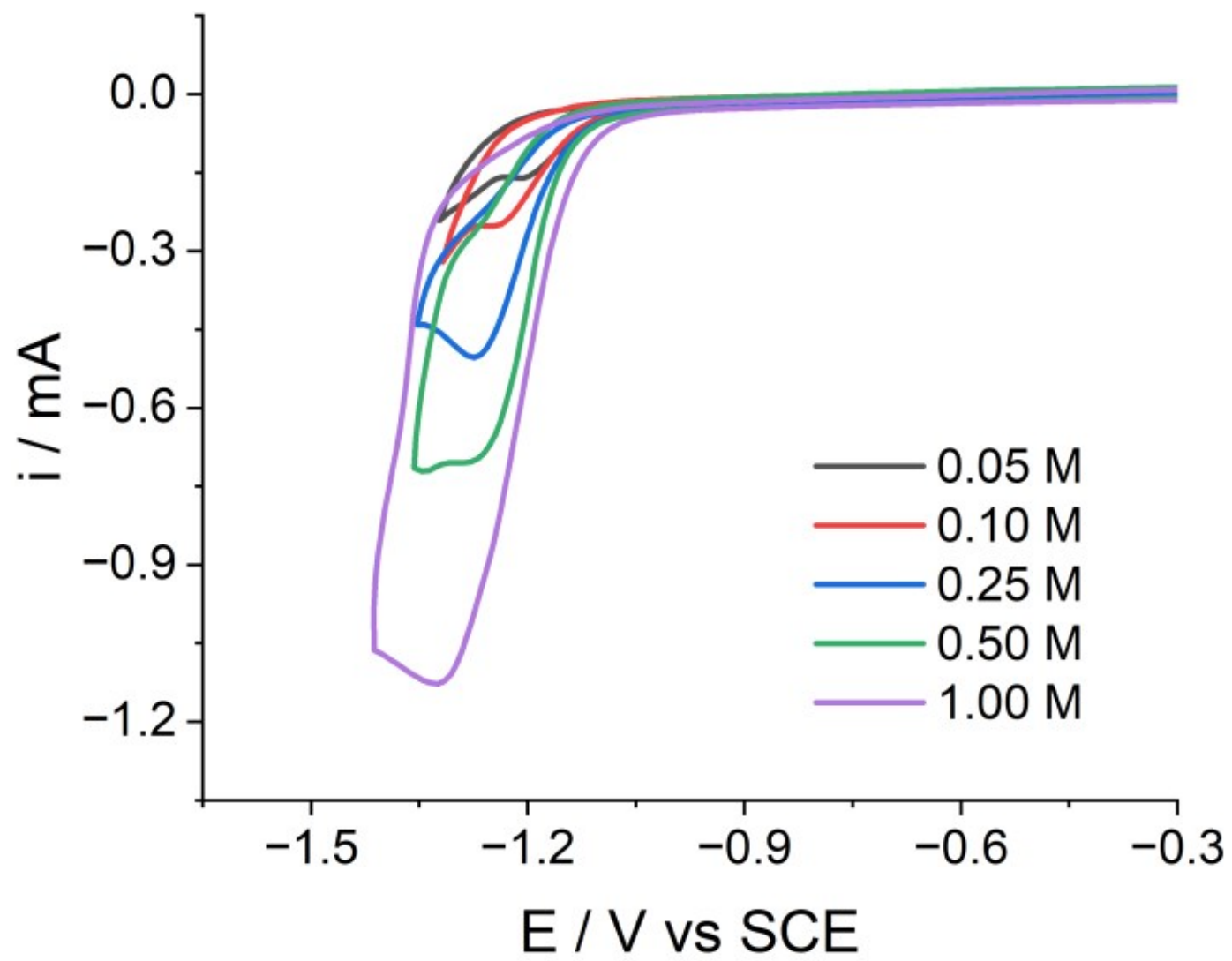

Figure S10. Comparison of CVs experiments recorded at a scan rate of 0.1 V/s in the presence of 50 mM $NaNO_2$ and 1 mM **CoL** at pH 7.4 in phosphate buffer solutions at different concentrations.

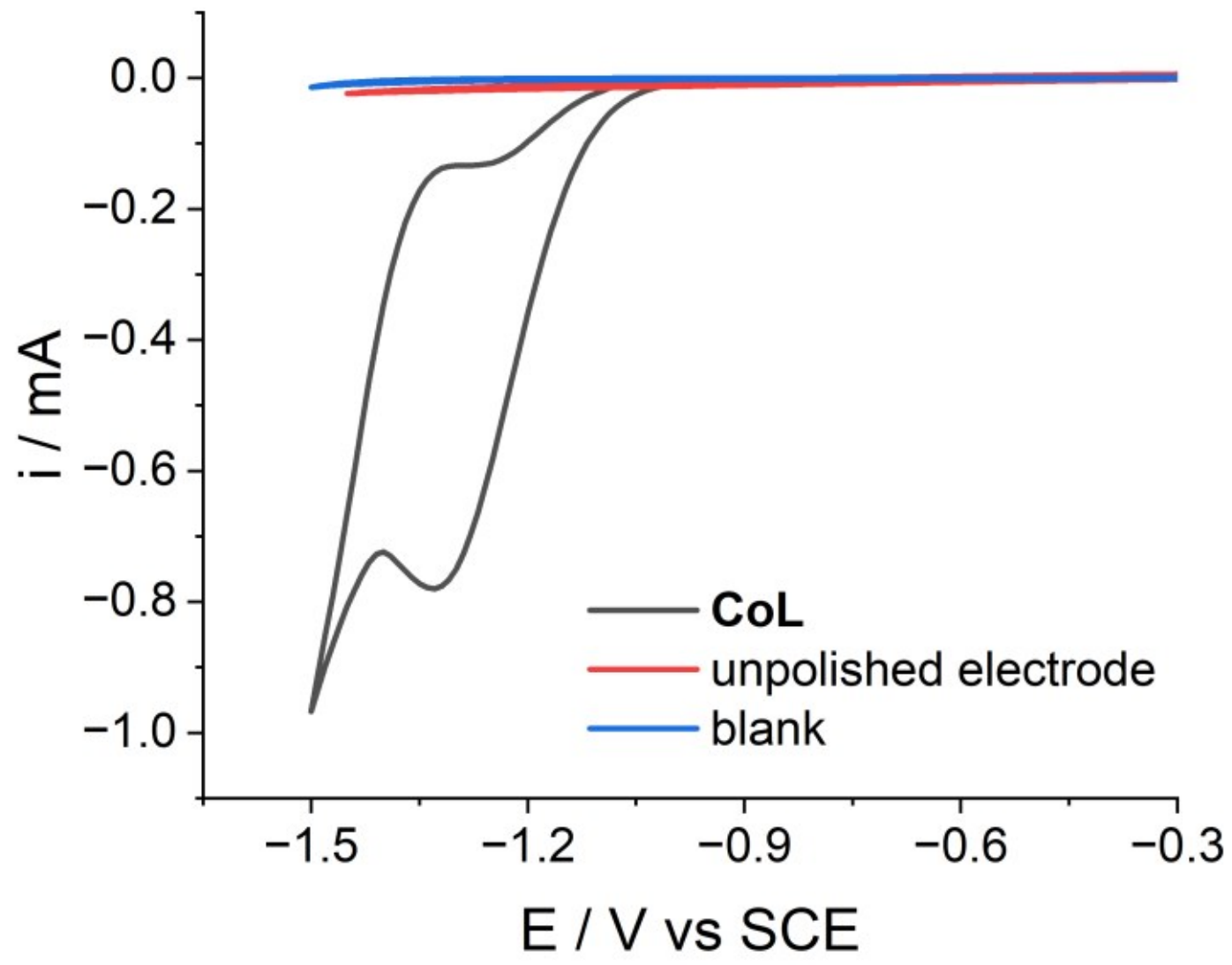

Figure S11. Comparison of CVs experiments recorded in 0.25 M phosphate buffer solution at pH 7 at a scan rate of 0.1 V/s in the presence of 50 mM $NaNO_2$ and 1 mM **CoL** (black trace), using the GC electrode previously employed with **CoL** but in the absence of **CoL** (unpolished electrode, red trace), and blank experiment using a polished GC electrode (blue trace).

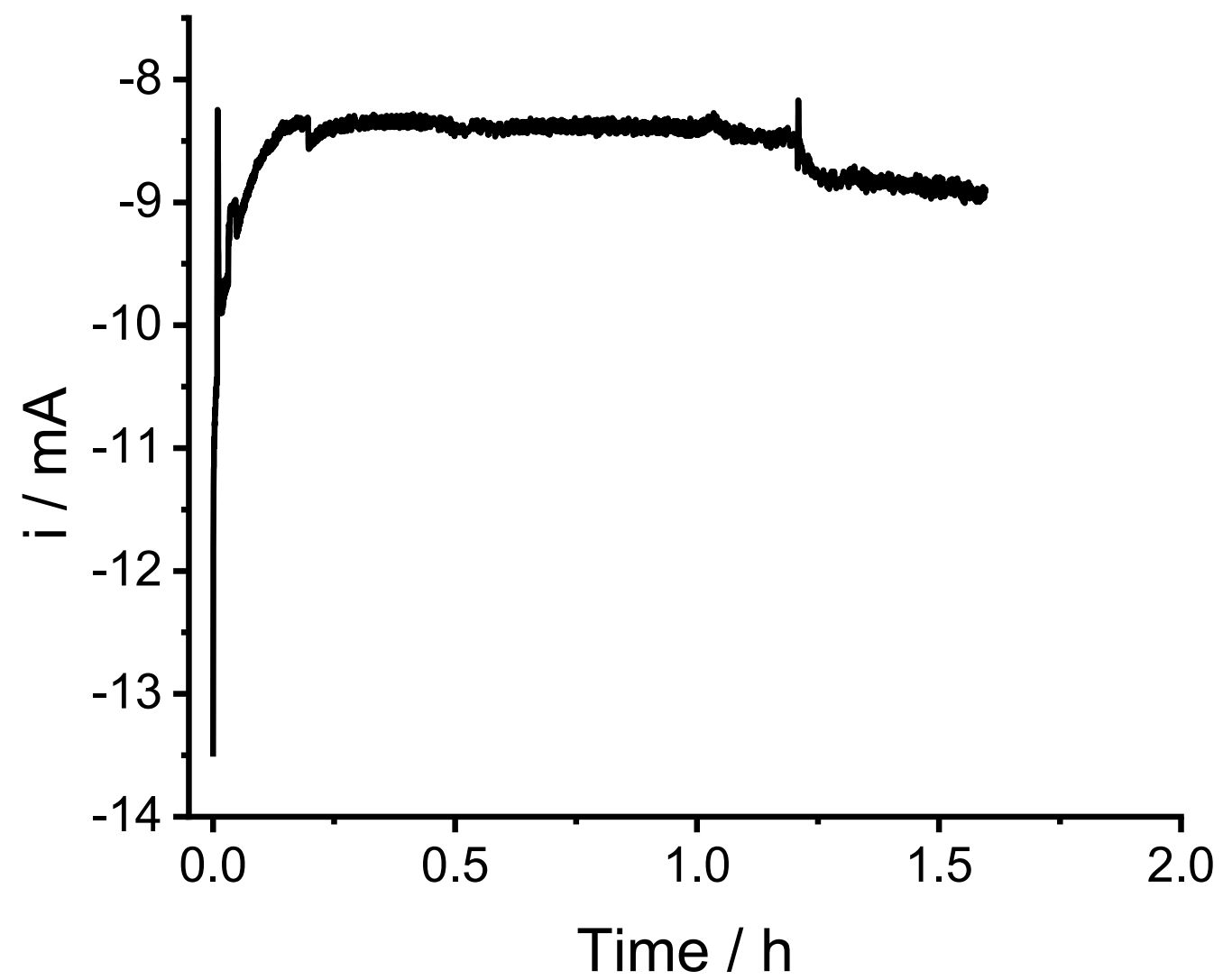

Figure S12. Chronoamperometry curve obtained at −1.4 V vs SCE on a 0.25 M phosphate buffer solution at pH 7 containing 1 mM **CoL** and 50 mM $NaNO_2$.

S2.1 TOF calculation

Estimation of the TOF was possible only at high phosphate buffer concentrations where an S-shaped profile was attained with plateau currents independent of scan rate for high scan rate values (see Figure S13 and S14). Under these conditions, the catalytic plateau current i_c can be described according to eq. S1,[S32,S33] where n = 6, F is the Faraday constant (96,485 C/mol), A is the electrode surface area (0.071 cm^2), C_{cat} is the catalyst concentration (0.001 M), and D is the diffusion coefficient (~$2\cdot10^{-6}$ cm^2/s). Since the exact value in water cannot be reliably determined from the Randles-Sevcik equation due to the irreversible nature of the Co(II)/Co(I) process (see above), the diffusion coefficient was approximated using the value in acetonitrile ($5\cdot10^{-6}$ cm^2/s)[S34] and the Stokes-Einstein inverse-viscosity scaling according to eq. S2, where η_{CH_3CN} = 0.37 mPa·s and η_{H_2O} = 0.89 mPa·s.

$$i_c = nFAC_{cat}D^{1/2}TOF^{1/2} \tag{S1}$$

$$D_{H_2O} \approx D_{CH_3CN}\frac{\eta_{CH_3CN}}{\eta_{H_2O}} \tag{S2}$$

Considering the catalytic plateau current values of –2.16 and –2.90 mA recorded at pH 7.4 at high scan rates in the presence of 50 mM $NaNO_2$ and 1 mM **CoL** (Figure S13 and S14), TOF values of 1380 and 2490 s^{-1} were estimated in 0.5 and 1 M phosphate buffer solution, respectively.

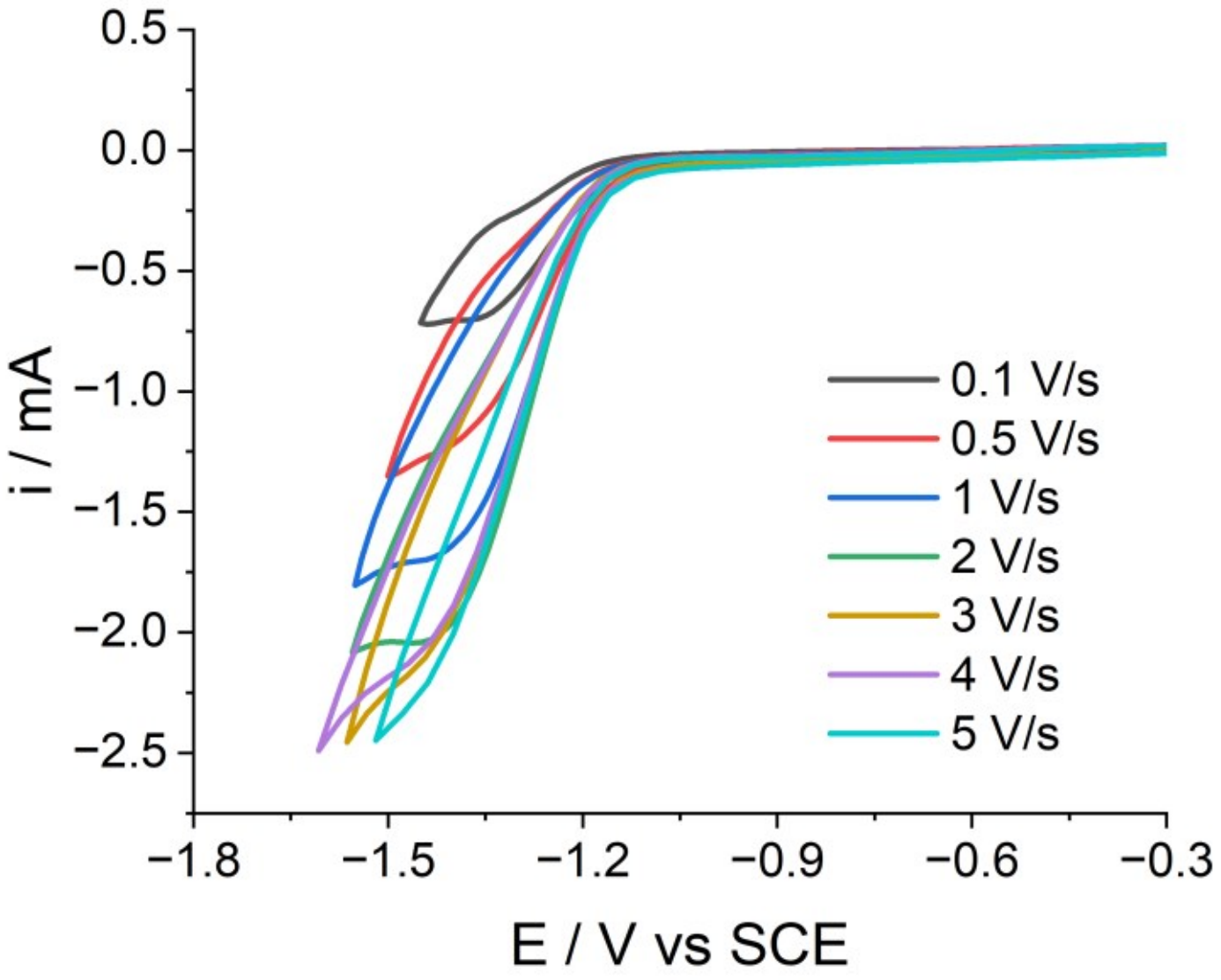

Figure S13. Comparison of CVs experiments at variable scan rates recorded at pH 7.4 in 0.5 M phosphate buffer solution in the presence of 50 mM $NaNO_2$ and 1 mM **CoL**.

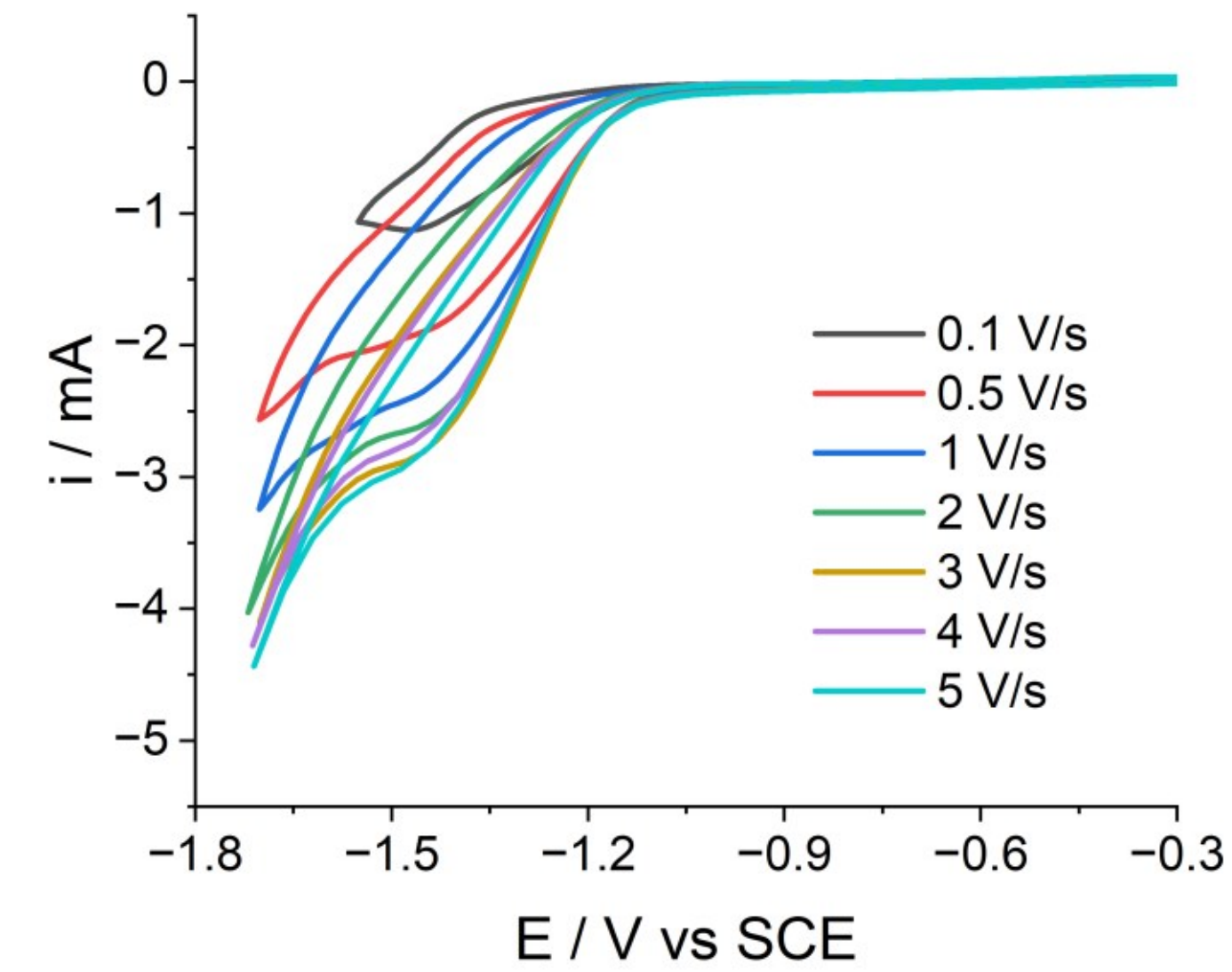

Figure S14. Comparison of CVs experiments at variable scan rates recorded at pH 7.4 in 1 M phosphate buffer solution in the presence of 50 mM $NaNO_2$ and 1 mM **CoL**.

S3. Light-driven catalysis

S3.1 Key performance indicators

The maximum turnover number (TON) was estimated according to eq. S3, where the n_{max} and n_{cat} are the moles of NH_3 and the moles of catalyst, respectively.

$$TON = \frac{n_{max}}{n_{cat}} \tag{S3}$$

The quantum yield (Φ) was calculated according to eq. S4, where r is the initial rate of NH_3 production ($mol \cdot s^{-1}$) calculated from the slope in the linear portion of the kinetic trace and φ is the absorbed photon flux ($Einstein \cdot s^{-1}$).

$$\Phi = \frac{r}{\varphi} \tag{S4}$$

The quantification of the absorbed photon flux (φ) was performed by employing the photoreaction between $[Ru(bpy)_3]Cl_2 \cdot 6H_2O$ and 9,10-diphenylanthracene (DPA) in aerated acetonitrile solution as an actinometer, following an established protocol.[S35] A 6 mL solution consisting of 0.4 mM $[Ru(bpy)_3]Cl_2 \cdot 6H_2O$ and 0.1 mM DPA in acetonitrile was prepared and its UV-Vis spectrum was recorded. Subsequently, the solution was subjected to a one-minute irradiation using the same light source and setup employed in the photochemical experiments. A new UV-Vis spectrum of the irradiated sample was then acquired. Both samples were diluted for the UV-Vis spectra measurements by mixing 1 mL of the sample with 2 mL of acetonitrile. The moles of DPA consumed were computed following eq. S5 where A_i is the absorbance at 372 nm before the irradiation, A_f is the absorbance at 372 nm after the irradiation, V is the volume of the sample (considering dilution) and ε_{372nm} = 11,100 $M^{-1}cm^{-1}$ is the attenuation coefficient of DPA at 372 nm in acetonitrile. A cuvette with a 1 cm path length was employed.

$$n\ DPA\ consumed = \frac{A_i - A_f}{\varepsilon_{372\ nm}} \times V \quad \text{(S5)}$$

The absorbed photon flux (φ) was calculated using eq. S6, where Φ_{ref} = 0.019 is the quantum yield of the actinometer and t = 60 s is the irradiation time.

$$\varphi = \frac{n\ DPA\ consumed}{\Phi_{ref}\ t} \quad \text{(S6)}$$

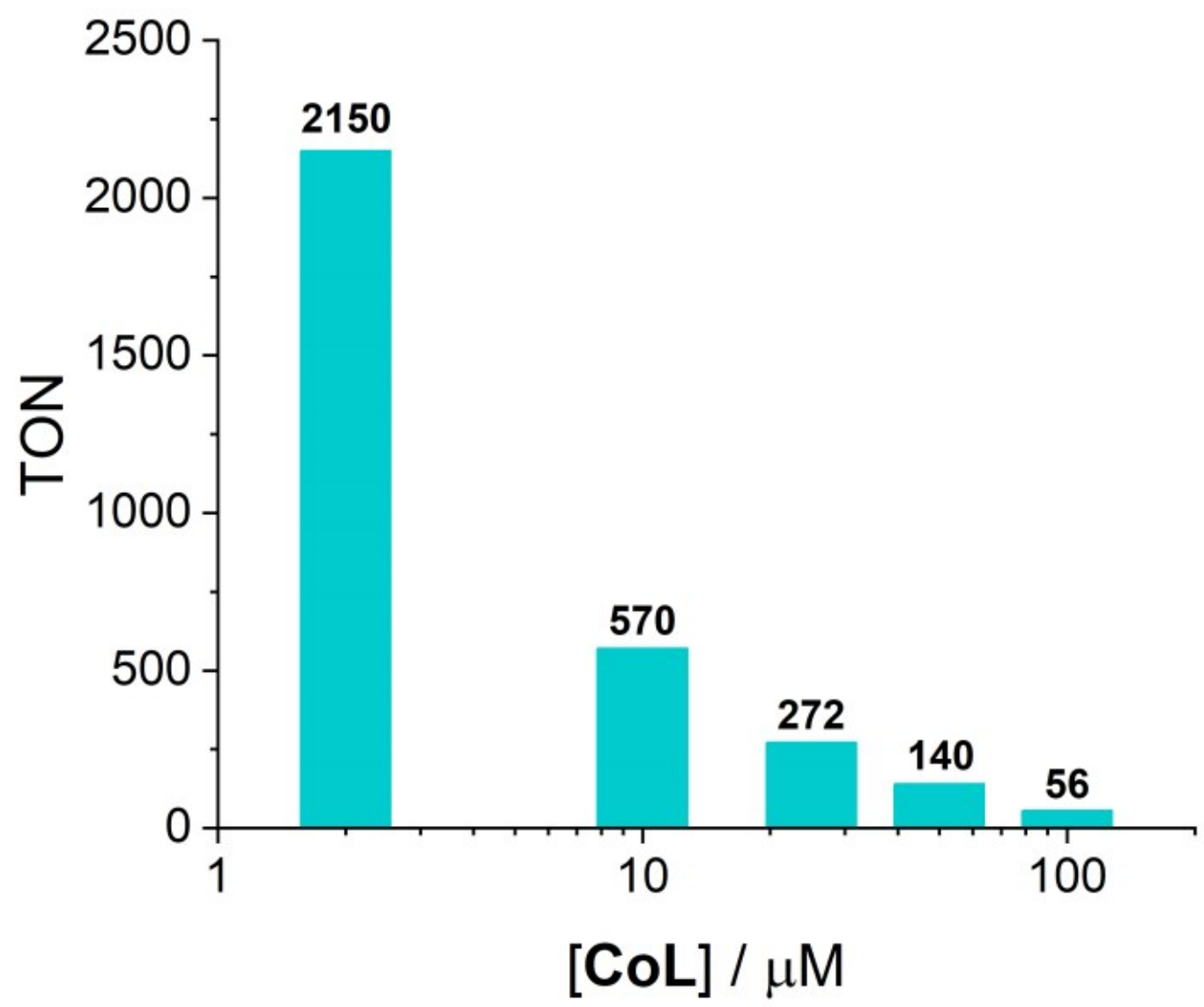

Figure S15. Maximum TONs obtained after 23 h irradiation (LED at 460 nm) of a 0.25 M phosphate buffer solution at pH 7 containing 0.5 mM $[Ru(bpy)_3]Cl_2 \cdot 6H_2O$, 0.1 M sodium ascorbate, 2-100 µM **CoL**, and 50 mM $NaNO_2$.

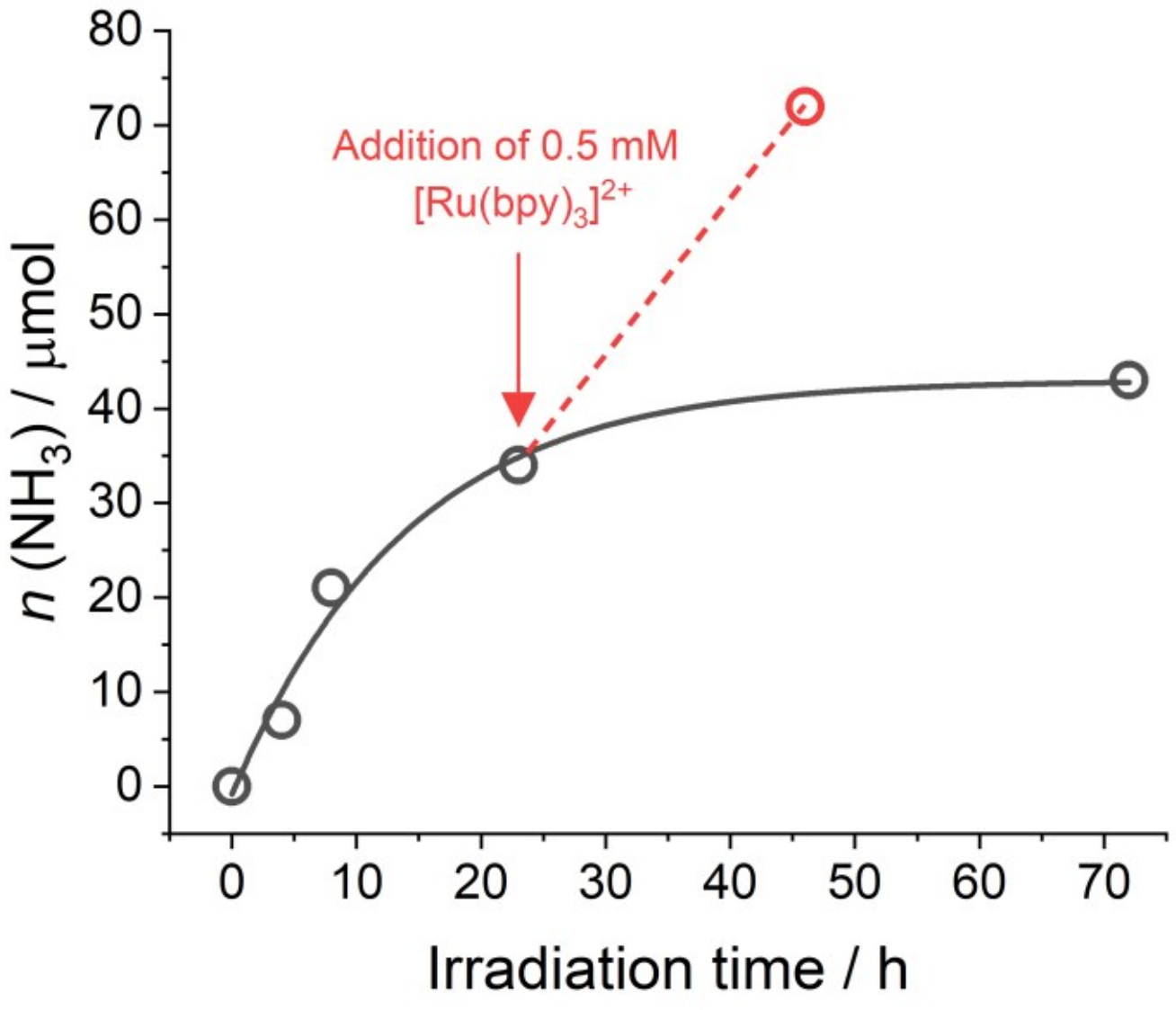

Figure S16. Resumption of the NH_3 production under irradiation (LED at 460 nm) of a 0.25 M phosphate buffer solution at pH 7 containing 0.5 mM $[Ru(bpy)_3]Cl_2{\cdot}6H_2O$, 0.1 M sodium ascorbate, 25 μM **CoL**, and 50 mM $NaNO_2$.

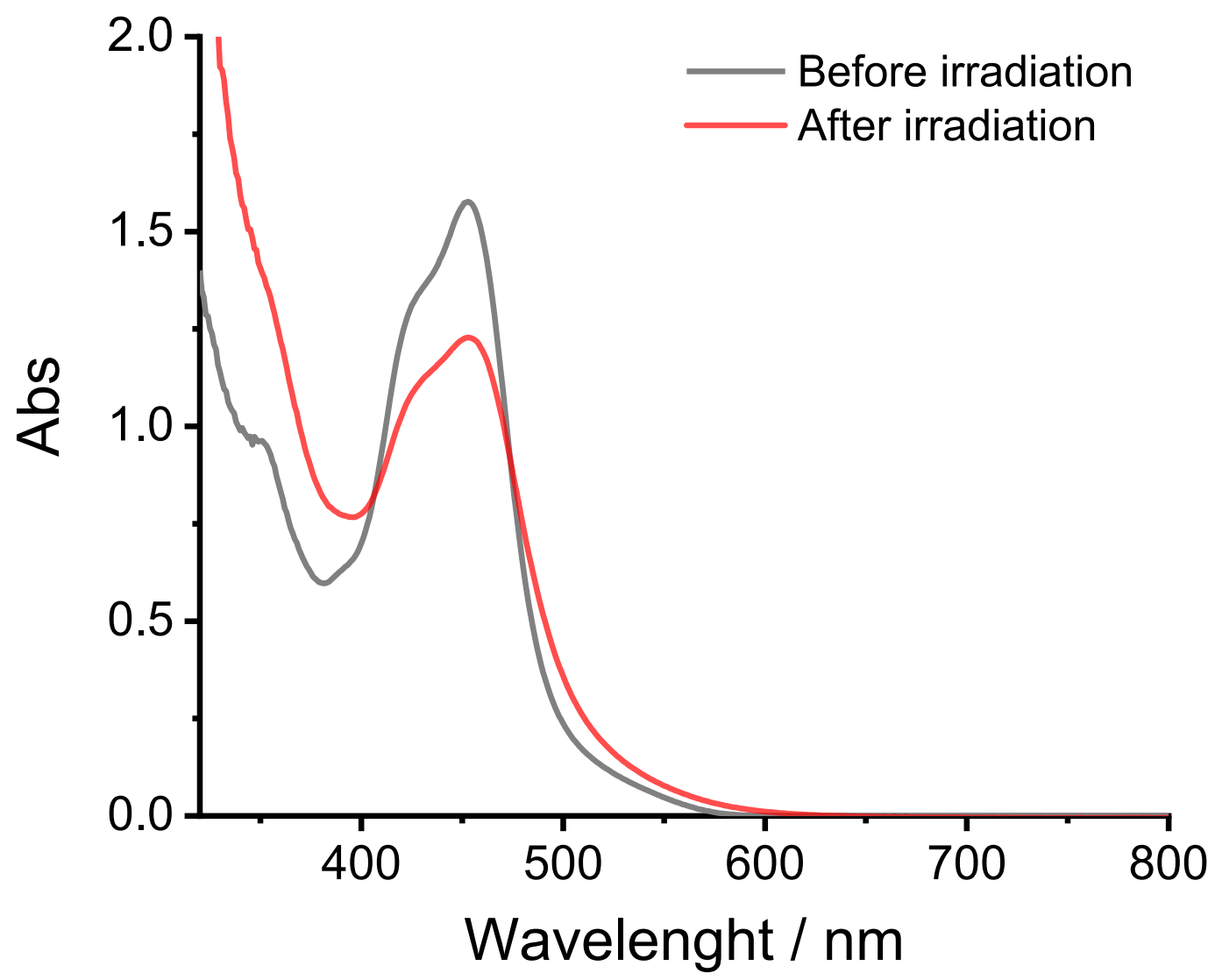

Figure S17. Absorption spectra before and after 72 h irradiation (LED at 460 nm) of a 0.25 M phosphate buffer solution at pH 7 containing 0.5 mM $[Ru(bpy)_3]Cl_2{\cdot}6H_2O$, 0.1 M sodium ascorbate, 25 μM **CoL**, and 50 mM $NaNO_2$.

Table S1. Effect of pH on ammonia production.[a]

pH	[CoL] / μM	NH_3 / μmol	TON
5.5	25	18.8	150
7	25	34.0	272
8.5	25	19.3	154

[a] After 23 h irradiation (LED at 460 nm) of 0.25 M phosphate buffer solution containing 0.5 mM $[Ru(bpy)_3]Cl_2 \cdot 6H_2O$, 0.1 M sodium ascorbate, and 50 mM $NaNO_2$.

Table S2. Effect of $NaNO_2$ concentration.[a]

$NaNO_2$ / mM	[CoL] / μM	NH_3 / μmol	TON
50	10	28.5	570
100	10	27.3	546
500	10	31.5	630
50	25	34.0	272
500	25	32.4	259

[a] After 23 h irradiation (LED at 460 nm) of 0.25 M phosphate buffer solution at pH 7 containing 0.5 mM $[Ru(bpy)_3]Cl_2 \cdot 6H_2O$, and 0.1 M sodium ascorbate.

Table S3. Effect of phosphate buffer concentration on ammonia production.[a]

P buffer / M	[CoL] / μM	NH_3 / μmol	TON
0.10	25	21.1	167
0.25	25	34.0	272
1.00	25	34.8	278

[a] After 23 h irradiation (LED at 460 nm) of phosphate buffer solutions at pH 7 containing 0.5 mM $[Ru(bpy)_3]Cl_2 \cdot 6H_2O$, 0.1 M sodium ascorbate, and 50 mM $NaNO_2$.

Table S4. Control experiments.[a]

Entry	Conditions	NH_3 / μmol
1	No light	-
2	No $[Ru(bpy)_3]Cl_2 \cdot 6H_2O$	-
3	No ascorbate	-
4	No **CoL**	0.4

[a] After 8 h irradiation (LED at 460 nm) of 0.25 M phosphate buffer solution at pH 7 containing 0.5 mM $[Ru(bpy)_3]Cl_2 \cdot 6H_2O$, 0.1 M sodium ascorbate, 25 μM **CoL**, and 50 mM $NaNO_2$.

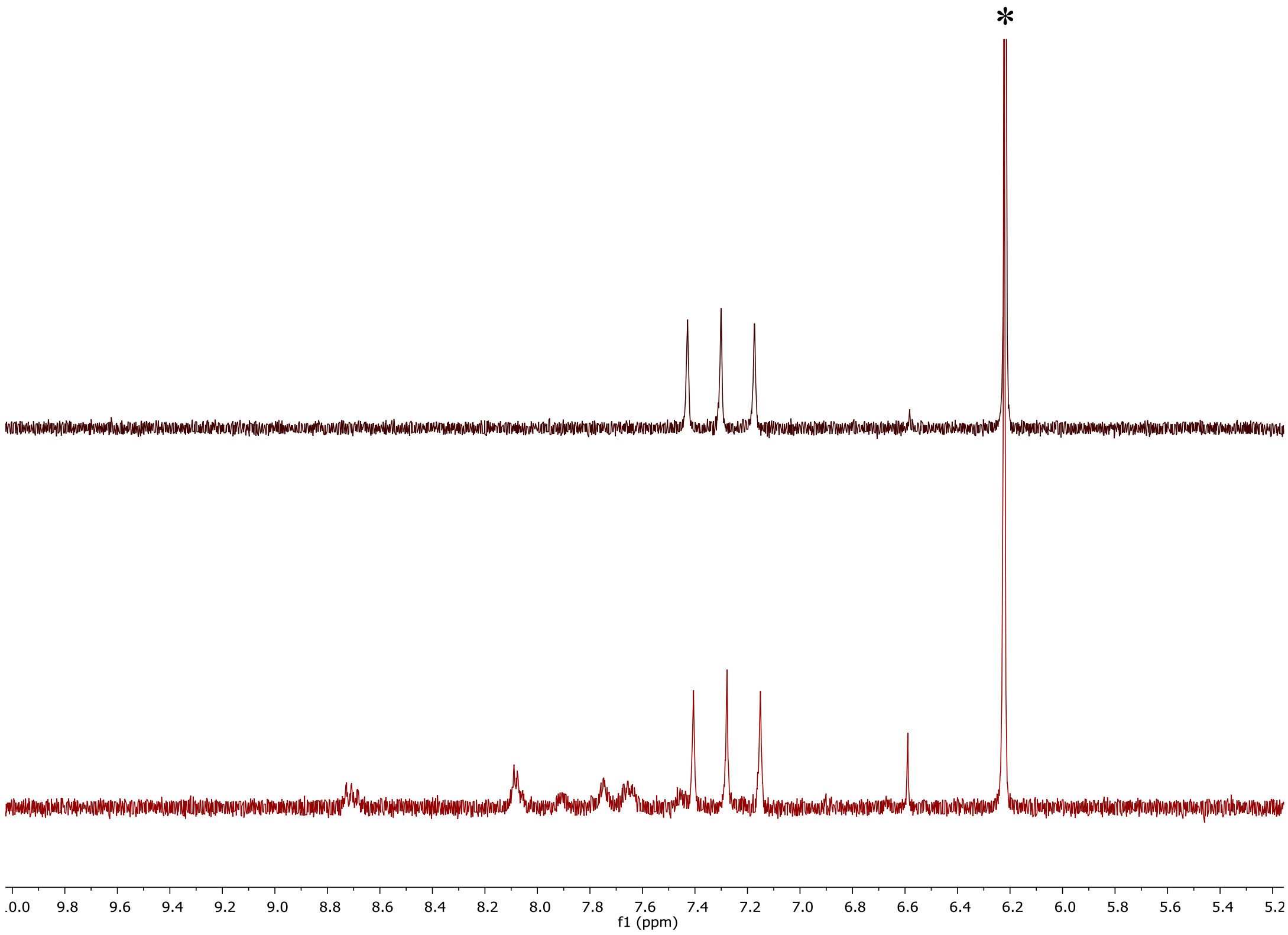

Figure S18. Comparison of ^{1}H-NMR spectra in DMSO-d_6 before (bottom) and after (top) vacuum transfer of the photolyzed solution (experimental conditions: 23 h irradiation with an LED at 460 nm, 0.25 M phosphate buffer at pH 7, 0.5 mM $[Ru(bpy)_3]Cl_2{\cdot}6H_2O$, 0.1 M sodium ascorbate, 25 μM **CoL**, and 50 mM $NaNO_2$), previously treated with 3 M NaOH (1.5 mL), into a 3 M HCl solution (4 mL). The quantitative analysis performed using the maleic acid standard (*) indicates a 100% transfer of the NH_3 photoproduct.

S4. Mechanistic investigation

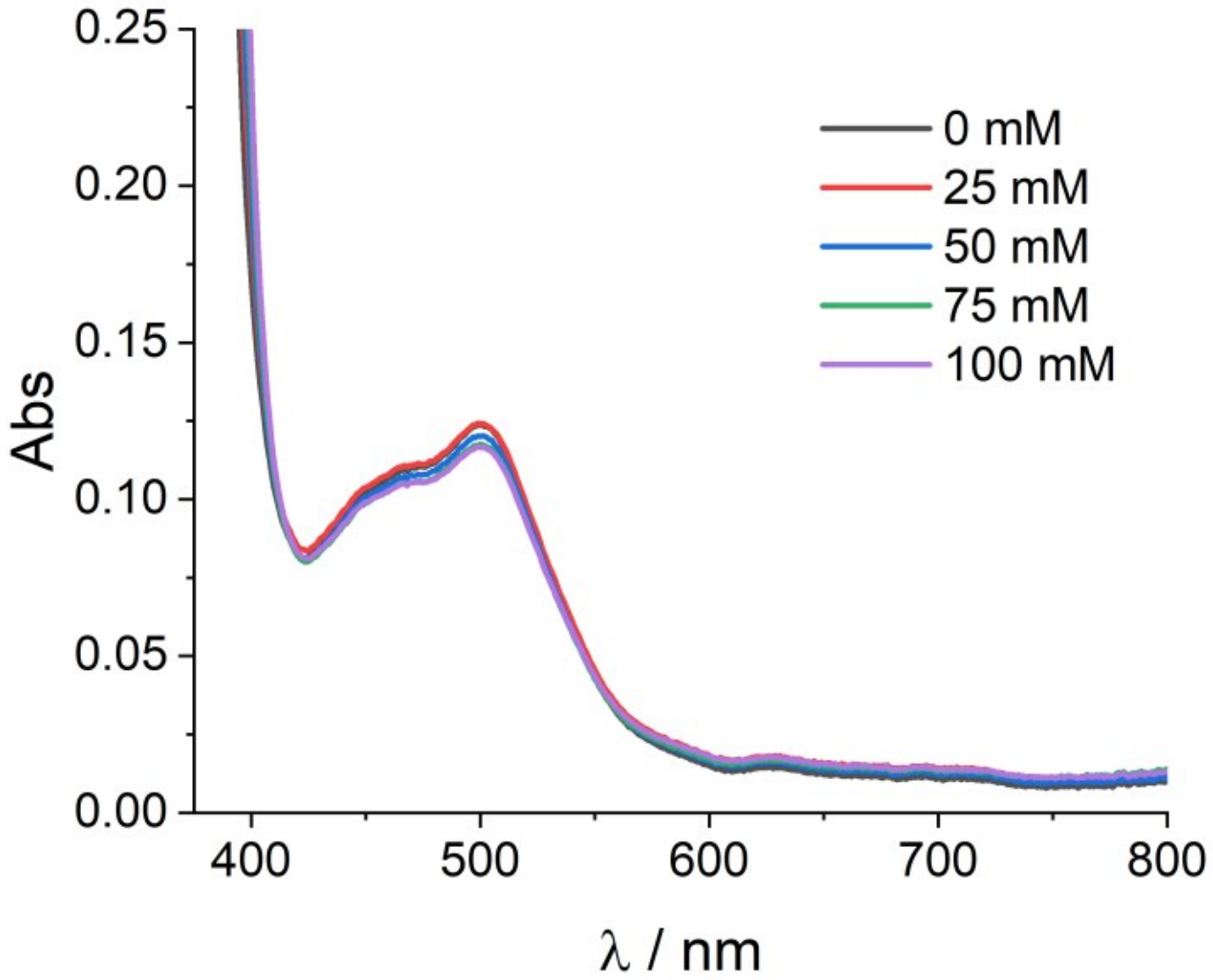

Figure S19. Comparison of absorption spectra of 4 mM **CoL** in 0.25 M phosphate buffer at pH 7 in the presence of variable amounts of $NaNO_2$.

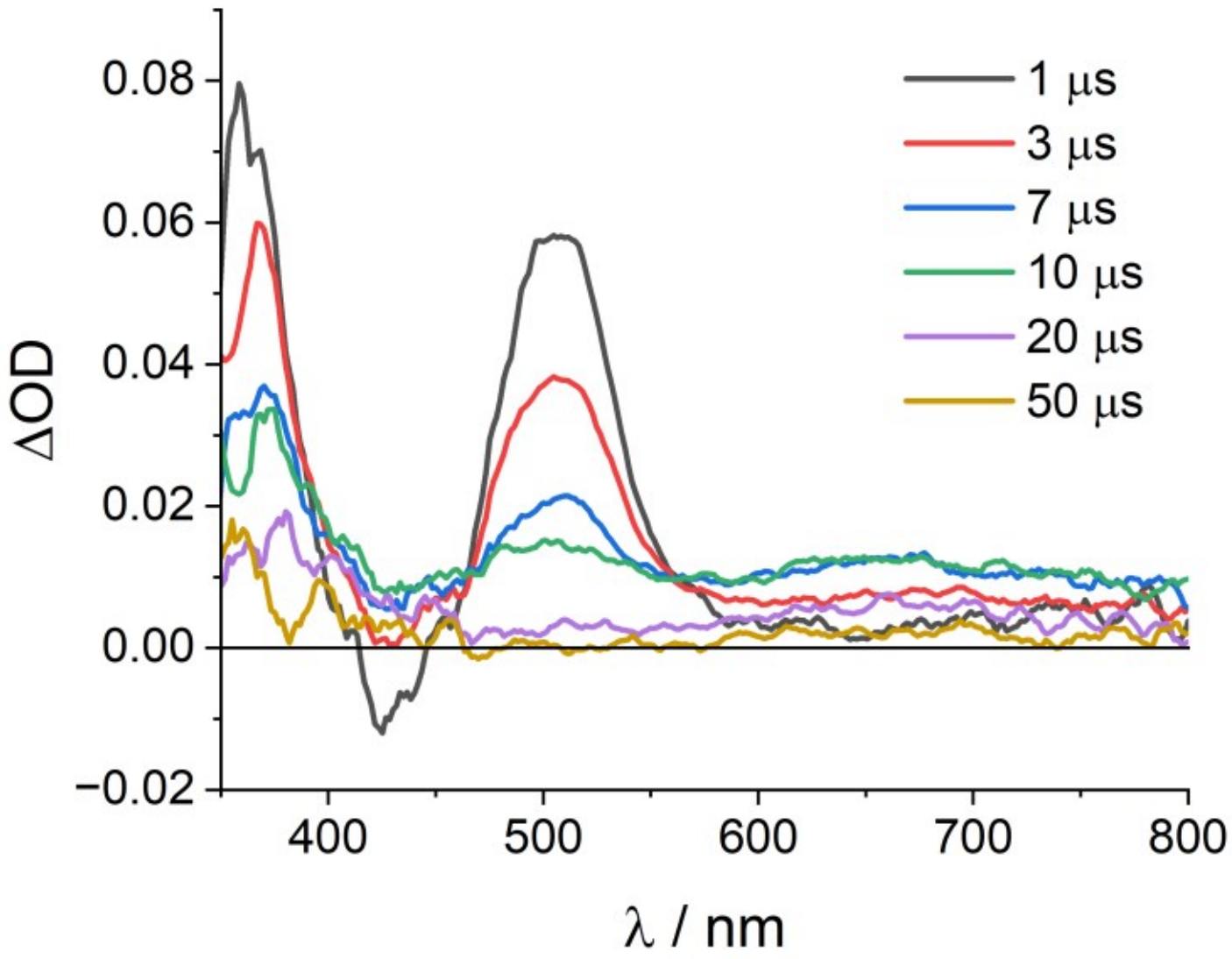

a)

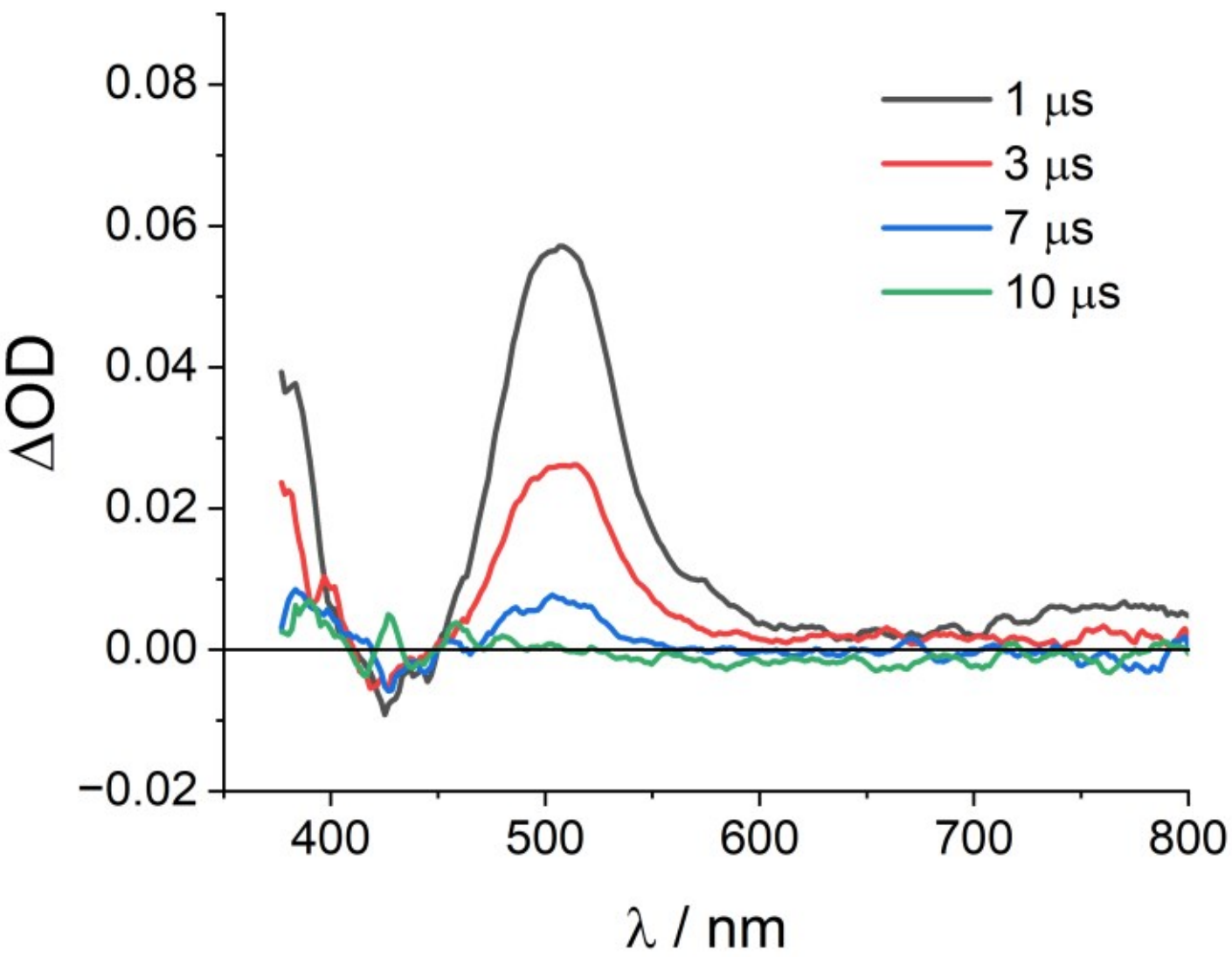

b)

Figure S20. Transient absorption spectra between 1-10 μs time delays measured by LFP (excitation at 532 nm) of an N_2-purged 0.25 M phosphate buffer solution at pH 7 containing 70 μM $[Ru(bpy)_3]Cl_2 \cdot 6H_2O$, 0.1 M sodium ascorbate, and 100 μM complex **CoL** a) in the absence and b) in the presence of 0.05 M $NaNO_2$.

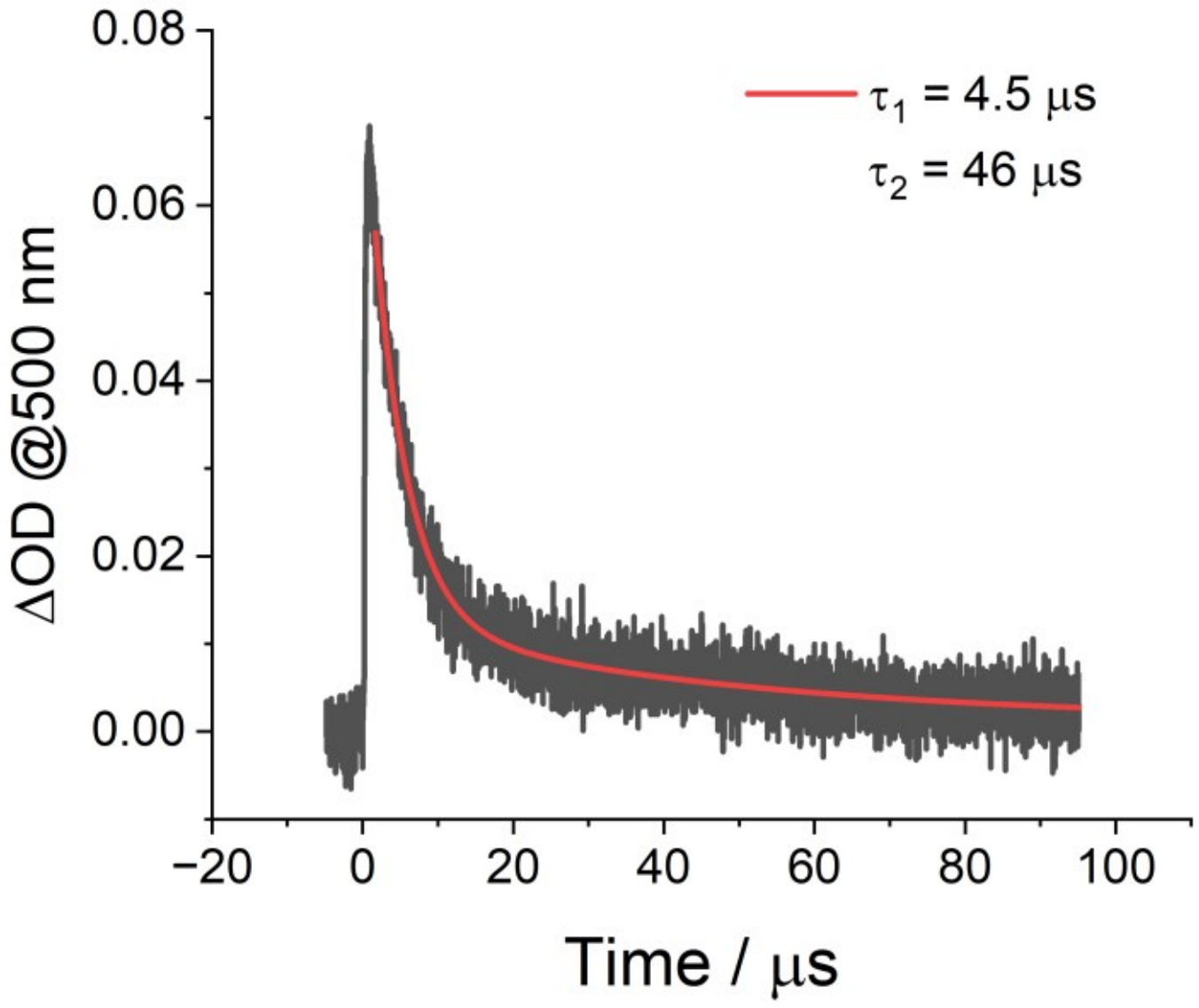

Figure S21. Kinetic trace at 500 nm measured by LFP (excitation at 532 nm) of an N_2-purged 0.25 M phosphate buffer solution at pH 7 containing 70 μM $[Ru(bpy)_3]Cl_2{\cdot}6H_2O$, 0.1 M sodium ascorbate, and 100 μM complex **CoL**, with related biexponential fitting: the first time component (τ = 4.5 μs) enables to estimate a bimolecular rate constant of $k = 2.2{\cdot}10^9$ $M^{-1}s^{-1}$ for the ET process from the reduced $[Ru(bpy)_3]^+$ to **CoL**, in perfect agreement with the value previously obtained under acidic conditions.[S35]

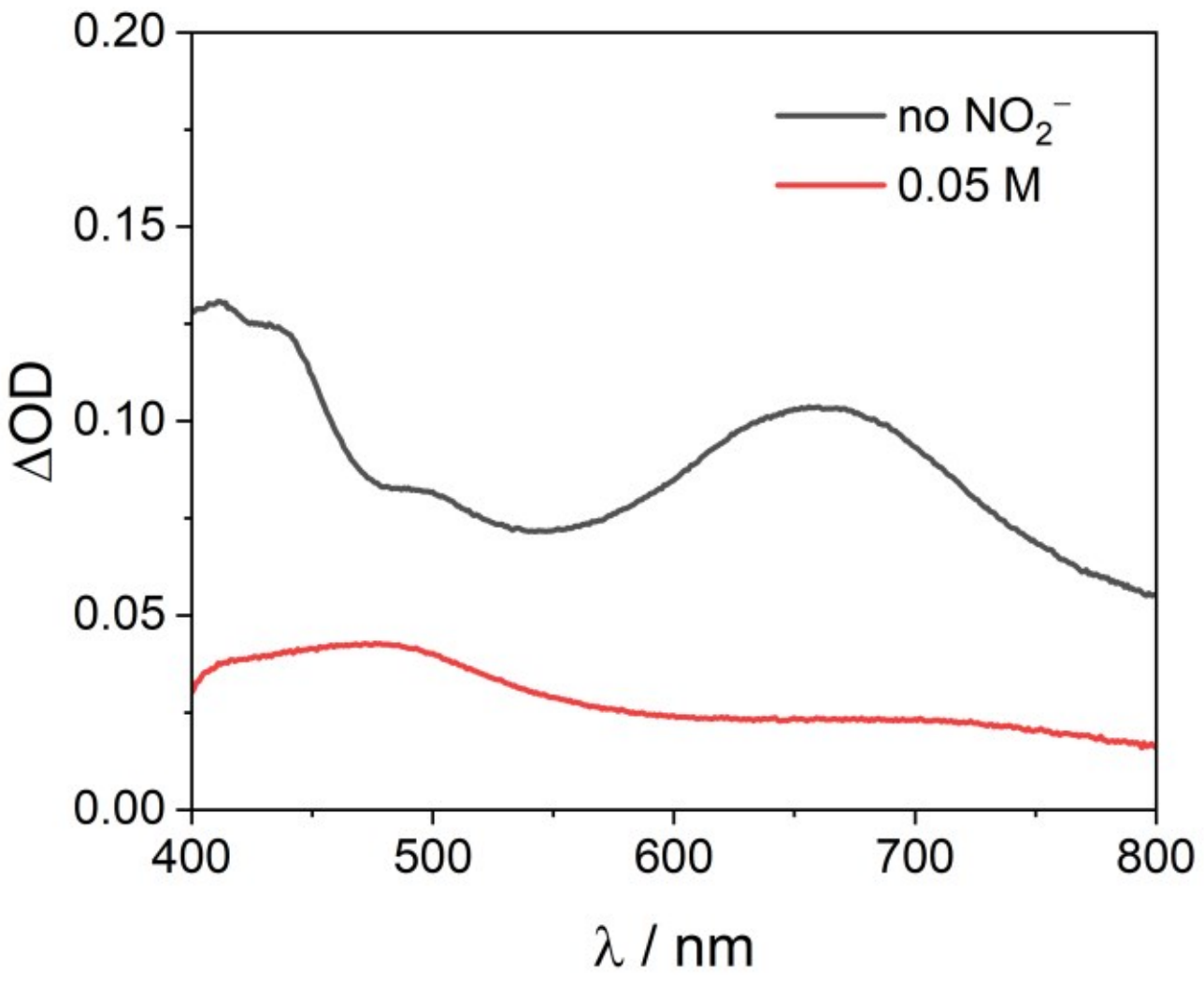

Figure S22. Differential spectra of the one-electron reduced **CoL** obtained by spectroelectrochemistry (applied potential = −1.4 V) of an acetonitrile solution (0.1 M $TBAPF_6$) containing 1 mM **CoL** in the absence and in the presence of 0.05 M $TBANO_2$.

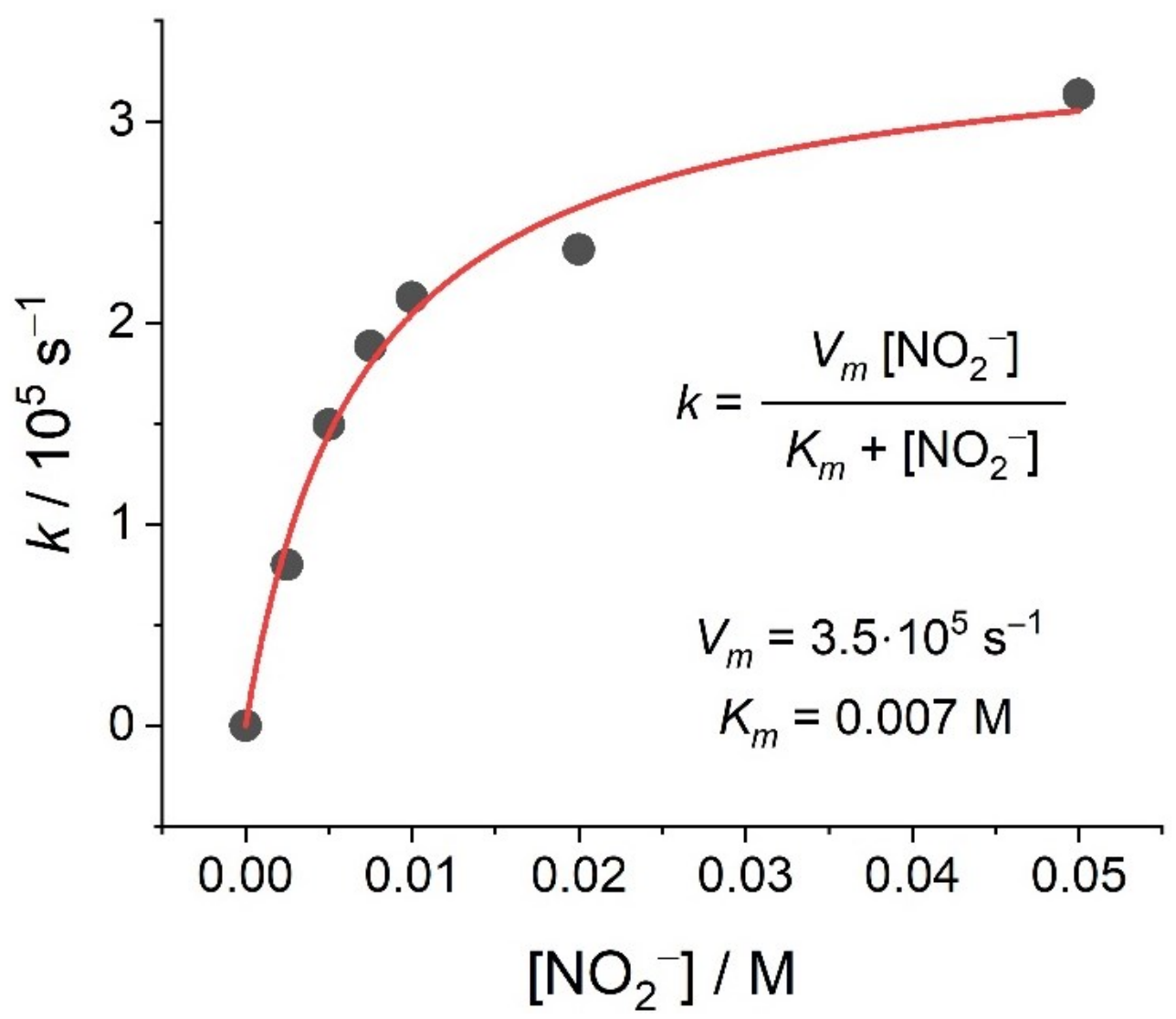

Figure S23. Plot of the observed rate *k* associated with the decay of the reduced **CoL** as a function of nitrite concentration with the corresponding fitting according to Michaelis-Menten saturation kinetics.

S4.1 Evans method

The magnetic moment (μ_{eff}) of **CoL** in its formal Co(II) and Co(I) oxidation states was calculated according to eqs S7 and S8, where χ_{mass} is the mass susceptibility (cm^3/g), MW is the molecular weight (g/mol), T is the temperature (K), Δf is the observed frequency difference (Hz) between the signal of the solvent in paramagnetic environment and in non-paramagnetic environment, f is the spectrometry frequency (Hz), m is the concentration of the paramagnetic substance (g/mL), and χ_0 is the mass susceptibility of the solvent in cm^3/g.[S36,S37]

$$\mu_{eff} = 2.83\sqrt{\chi_{mass} \cdot MW \cdot T} \quad \text{(S7)}$$

$$\chi_{mass} = \frac{3\Delta f}{4\pi f m} + \chi_0 \quad \text{(S8)}$$

The number of unpaired electrons was estimated comparing the experimental value of μ_{eff} (BM) with the theoretical value of the spin-only μ_{eff} calculated based on the number of unpaired electrons (n), according to eq S9.

$$\mu_{eff} = \sqrt{n(n+2)} \quad \text{(S9)}$$

In the adopted experimental conditions, MW = 709 g/mol, T = 300 K, f = 500.106 Hz, and χ_0 = $-6.30 \cdot 10^{-7}$ cm^3/g, the measured values of Δf were 425 Hz and 269 Hz before (Co(II)) and after (Co(I)) reduction, respectively (Figure S24). These values correspond to an μ_{eff} = 4.07 BM for Co(II) and μ_{eff} = 3.13 BM for Co(I), in good agreement with expected values of 3.87 BM and 2.83 BM calculated with eq S9 for a system with 3 and 2 unpaired electrons, respectively, consistent with a quartet and triplet spin multiplicity, respectively. The paramagnetic nature of both **CoL** and its reduced form was further confirmed upon registration of the NMR spectra above 15 ppm, which shows typical signals expected for paramagnetic compounds (Figure S25).

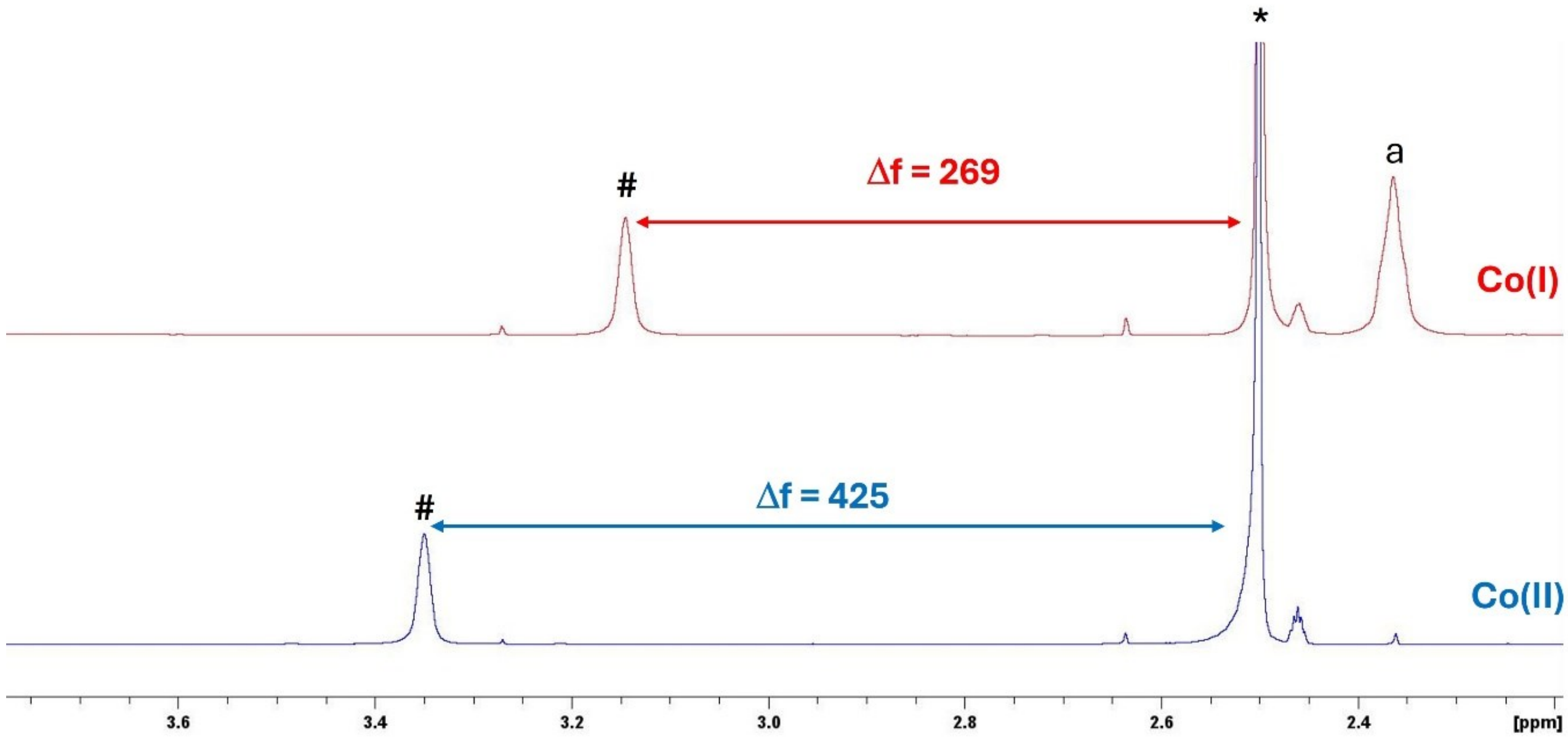

Figure S24. ^{1}H-NMR (500 MHz, d^6-DMSO) of a solution of **CoL** before (blue) and after (red) chemical reduction. # = DMSO signal in paramagnetic environment; * = DMSO signal in non-paramagnetic environment (sealed coaxial tube); a = THF; Δf = frequency shift (Hz).

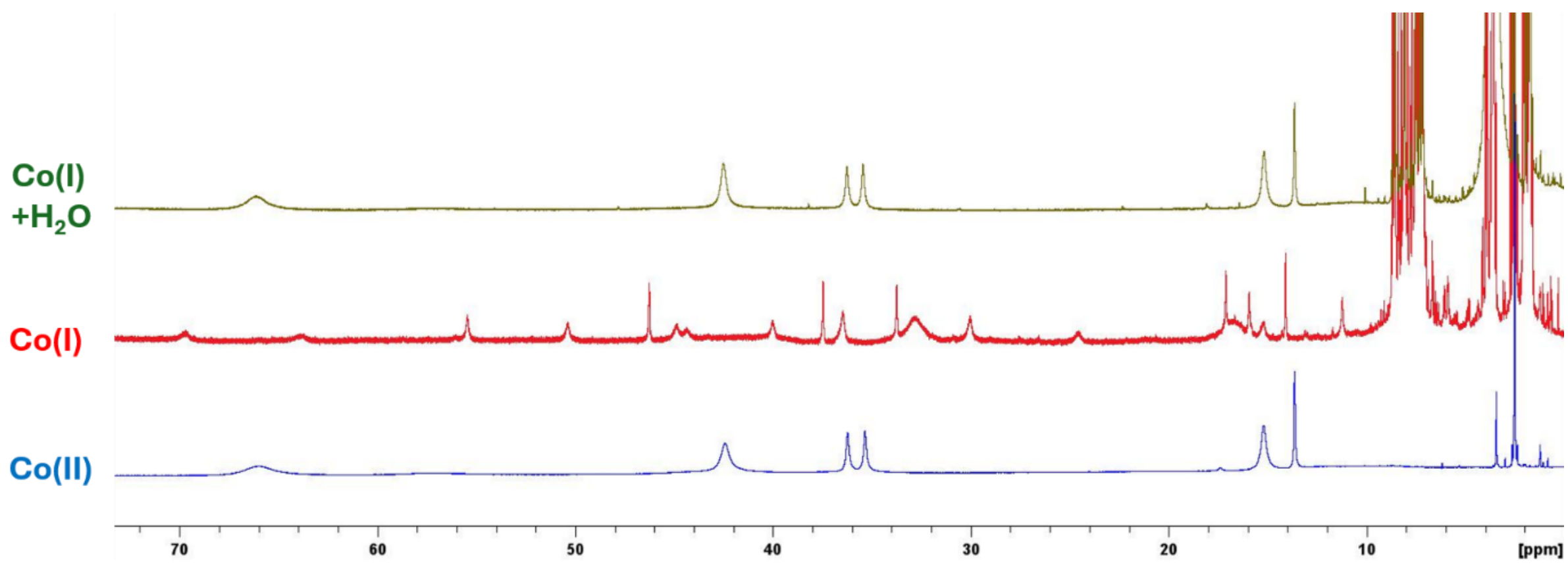

Figure S25. ^{1}H-NMR (500 MHz, d^6-DMSO) of a solution of **CoL** before (blue) and after (red) chemical reduction, and after addition of 0.1 mL H_2O to the reduced system (green). For this latter, a spectrum superimposable to Co(II) was obtained, in agreement with its reactivity with protons.[S1,S34]

S5. Computational studies

Scheme S1. Detailed catalytic mechanism for NO_2^-RR to NH_3 by **CoL** along with optimized structures of intermediates: potentials are reported in blue, free energy changes at pH 7 in red.

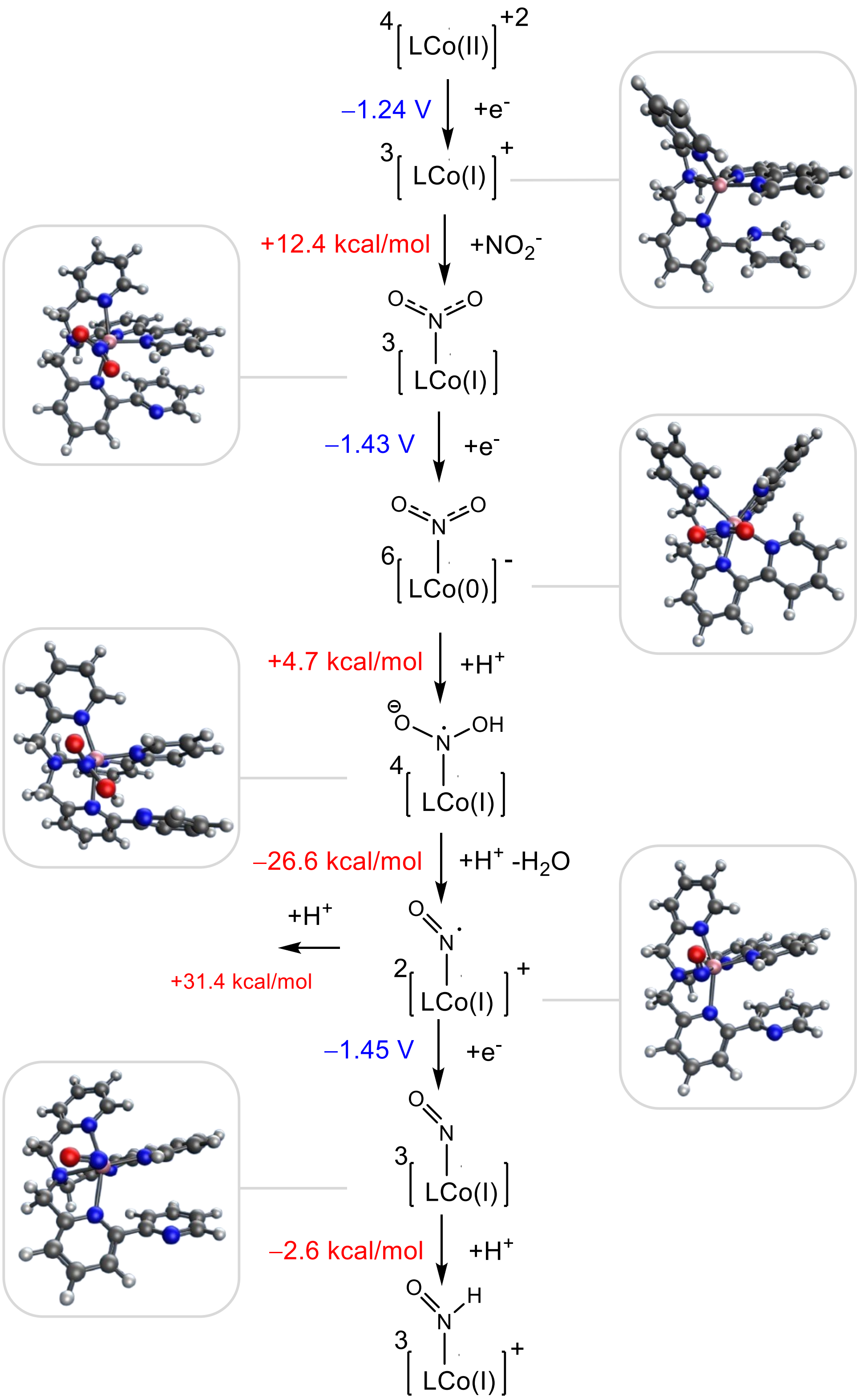

(continues on the next page)

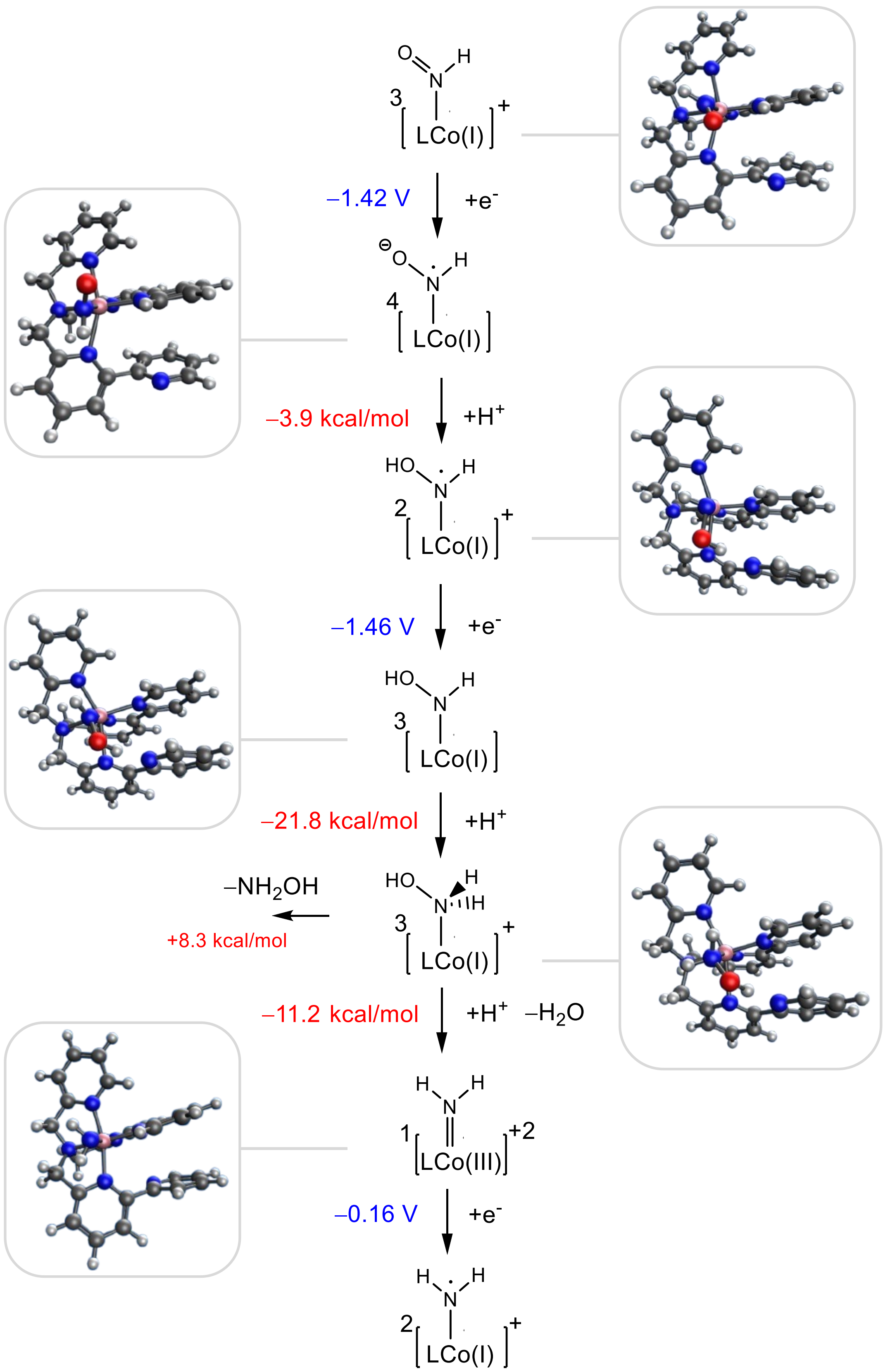

(continues on the next page)

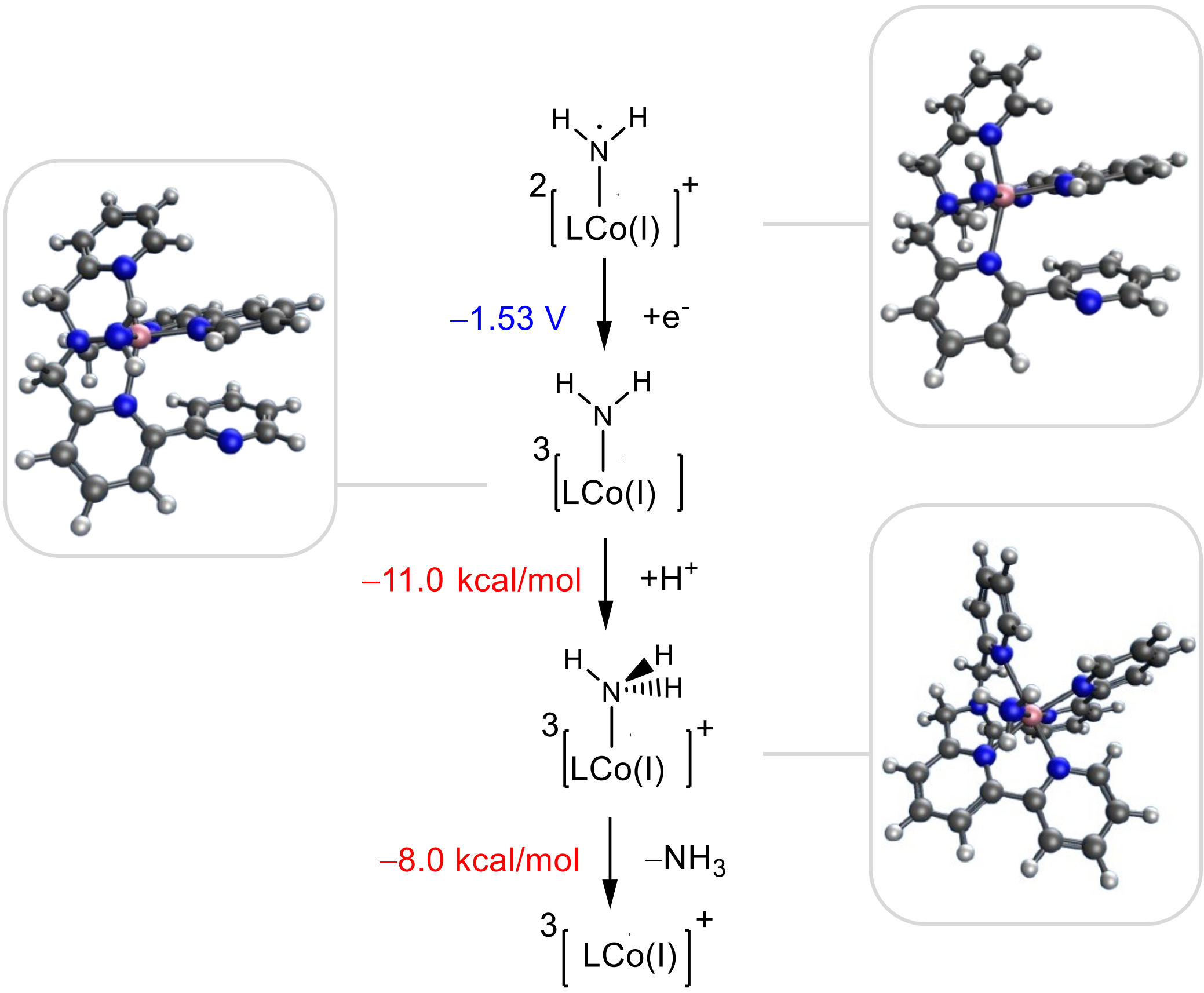

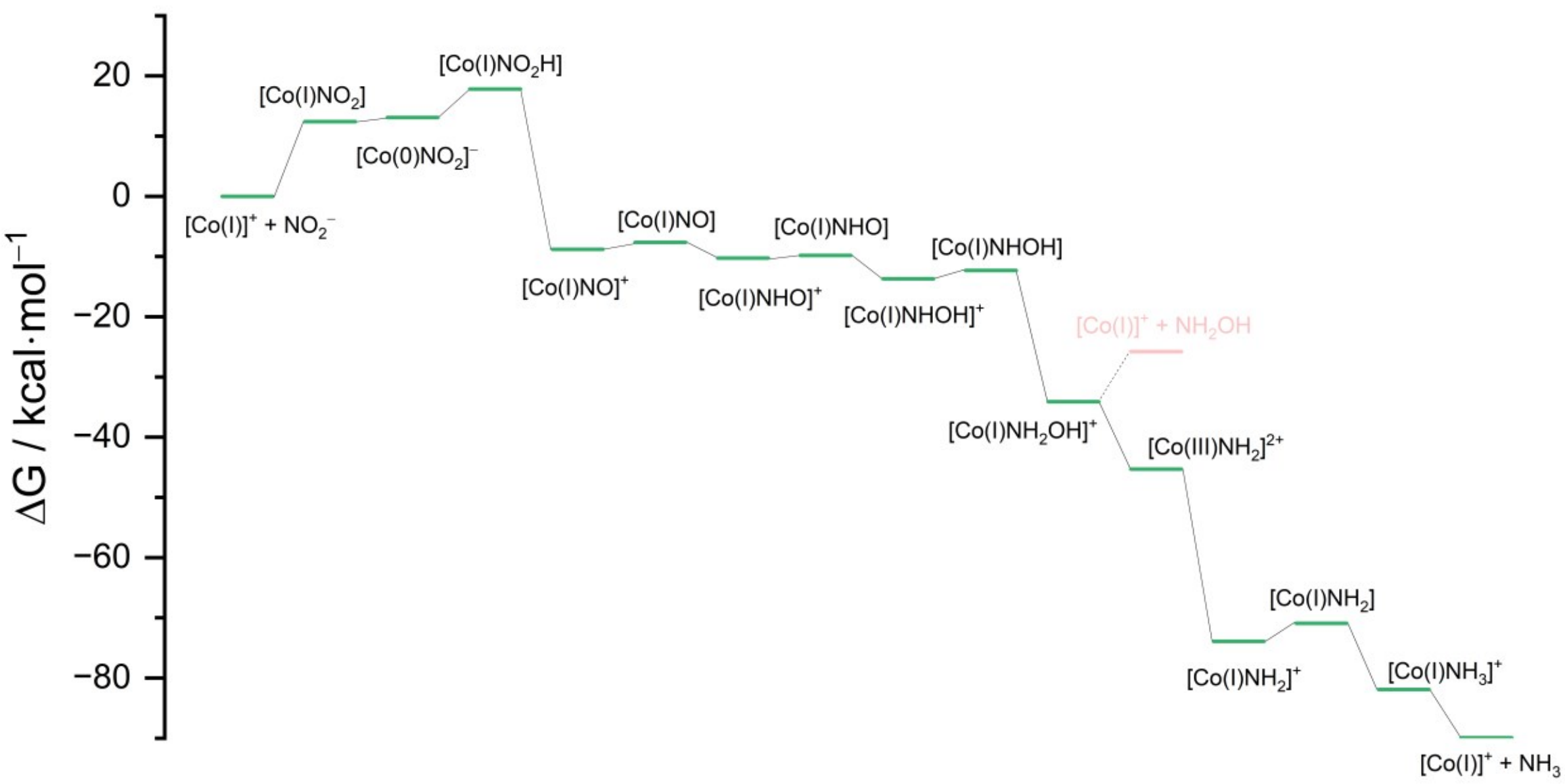

Figure S26. Free energy level diagram for NO_2^- reduction to NH_3 by **CoL** in aqueous solution: free energy changes have been determined at an applied potential of −1.4 V vs SCE and pH 7.

Table S5. Spin-crossover energies, in kcal·mol^{-1}, from low-spin to high-spin state of all analyzed molecular structures with an even number of electrons at the B3LYP*/def2-TZVPD/COSMO-RS level of theory (the negative value indicates that the high spin is more stable). The minimum energy geometries of each complex in each respective spin states were consistently utilized.

**Structure**	**Triplet – Singlet**	**Quintet – Singlet**	**Ground state**
$[LCo]^{+}$	–10.8	–3.6	triplet
$[LCo\text{-}NO_2]$	–13.0	–8.3	triplet
[LCo-NO]	–10.3	–3.3	triplet
$[LCo\text{-}(NH)O]^{+}$	–38.1	–31.1	triplet
[LCo-(NH)OH]	–11.4	–8.67	triplet
$[LCo\text{-}(NH_2)OH]^{+}$	–16.4	–5.4	triplet
$[LCo\text{-}NH_2]^{2+}$	4.2	13.8	singlet
$[LCo\text{-}NH_2]$	–8.6	–4.3	triplet
$[LCo\text{-}NH_3]$	–0.8	2.0	triplet

Table S6. Spin-crossover energies, in kcal·mol^{-1}, from low-spin to high-spin state of all analyzed molecular structures with an odd number of electrons at the B3LYP*/def2-TZVPD/COSMO-RS level of theory (the negative value indicates that the high spin is more stable). The minimum energy geometries of each complex in each respective spin states were consistently utilized.

**Structure**	**Quartet – Doublet**	**Sextet – Doublet**	**Ground state**
$[LCo]^{2+}$	–13.5	49.5	quartet
$[LCo\text{-}NO_2]^{-}$	1.0	–0.5	sextet
$[LCo(I)\text{-}NO_2H]$ [a]	N/A	N/A	quartet [b]
$[LCo\text{-}NO]^{+}$	10.8	19.4	doublet
[LCo-(NH)O]	–1.4	6.4	quartet
$[LCo\text{-}(NH)OH]^{+}$	4.6	23.2	doublet
$[LCo\text{-}NH_2]^{+}$	2.7	37.8	doublet

[a] No stable geometry with doublet multiplicity could be determined due to spontaneous elimination of hydroxide within geometry optimizations; [b] the spin-crossover energy from the sextet to the quartet state was calculated as +5.5 kcal·mol^{-1}.

Table S7. Absolute free energies of different conformers **A1-A6** associated with the Co(I) complex, in kcal·mol^{-1}, calculated at the B3LYP/def2-TZVPD/COSMO-RS level of theory, and free energies for the transition from the hexacoordinated structure **A6** in the same spin state, in kcal·mol^{-1}. The asterisk (*) denotes the minimum energy geometry, as drawn in Scheme S1.

Structure	Multiplicity	E_{abs} / kcal·mol^{-1}	E_{rel} / kcal·mol^{-1}
**A1** (κ^5)	singlet	–8.5	–9.8
	triplet	–25.0	–0.6
**A2** (κ^5)	singlet	–7.9	–9.2
	triplet	–25.3*	–0.9
**A3** (κ^4)	singlet	+2.2	+0.9
	triplet	–11.8	+12.5
**A4** (κ^4)	singlet	–6.0	–7.3
	triplet	–6.8	+17.5
**A5** (κ^4)	singlet	–9.0	–10.3
	triplet	–17.5	+6.9
**A6** (κ^6)	singlet	+1.3	0.0
	triplet	–24.4	0.0

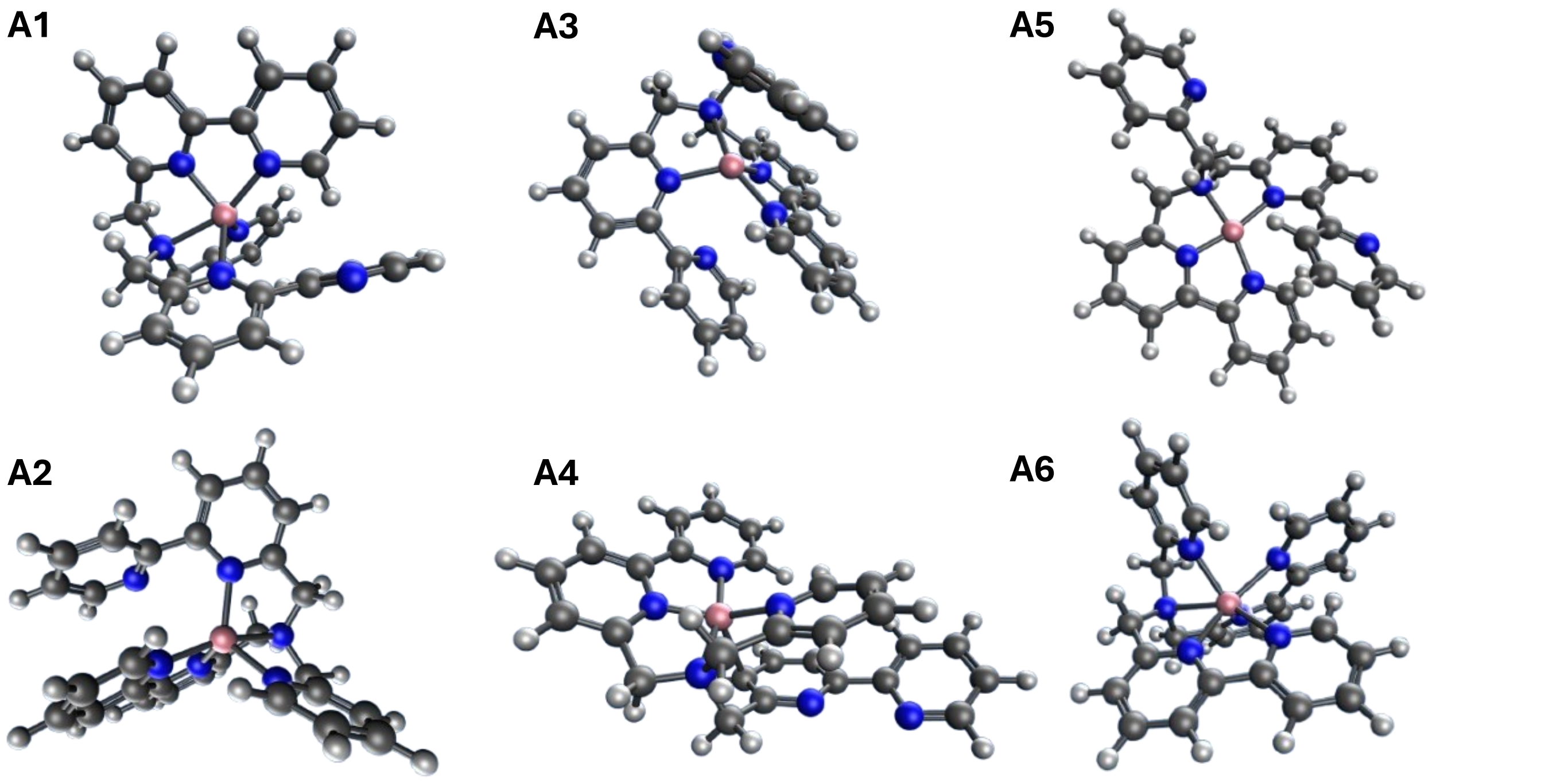

S6. References of the Supporting Information

S1 F. Lucarini, M. Pastore, S. Vasylevskyi, M. Varisco, E. Solari, A. Crochet, K. M. Fromm, F. Zobi, A. Ruggi, *Chem. Eur. J.* **2017**, *23*, 6768–6771.

S2 Bogdanovic, B.; Liao, S. T.; Mynott, R.; Sclichte, K.; Westeppe, U. *Chem. Ber.* **1984**, *117*, 1378-1392.

S3 Balasubramani, S. G., et al. *J. Chem. Phys.* **2020**, *152*, 184107.

S4 Grimme, S.; Antony, J.; Ehrlich, S.; Krieg, H. *J. Chem. Phys.* **2010**, *132*, 154104.

S5 Grimme, S.; Ehrlich, S.; Goerigk, L. *J. Comp. Chem.* **2011**, *32*, 1456-1465.

S6 Becke, A. D.; Johnson, E. R. *J. Chem. Phys*. **2005**, *122*, 154101.

S7 Johnson, E. R.; Becke, A. D. *J. Chem. Phys*. **2005**, *123*, 024101.

S8 Johnson, E. R.; Becke, A. D. *J. Chem. Phys*., **2006**, *124*, 174104.

S9 Becke, A. D. *Phys. Rev. A* **1988**, *38*, 3098-3100.

S10 Perdew, J. P. *Phys. Rev. B* **1986**, *33*, 8822-8824.

S11 Slater, J. C. *Phys. Rev.* **1951**, *81*, 385-390.

S12 Vosko, S. H.; Wilk, L.; Nusair, M. *Can. J. Phys.* **1980**, *58*, 1200-1211.

S13 Lee, C.; Yang, W.; Parr, R. G. *Phys. Rev. B* **1988**, *37*, 785-789.

S14 Reiher, M.; Salomon, O.; Artur Hess, B. *Theor. Chem. Acc*. **2001**, *107*, 48-55.

S15 Reiher, M. *Inorg. Chem*. **2002**, *41*, 6928-6935.

S16 Salomon, O.; Reiher, M.; Hess, B. A. *J. Chem. Phys.* **2002**, *117*, 4729-4737.

S17 Reimann, M.; Kaupp, M. *J. Chem. Theory Comput.* **2022**, *18*, 7442-7456.

S18 Reimann, M.; Kaupp, M. *J. Chem. Theory Comput.* **2023**, *19*, 97-108.

S19 Stephens, P. J.; Devlin, F. J.; Chabalowski, C. F.; Frisch, M. J. *J. Phys. Chem.* **1994**, *98*, 11623-11627.

S20 Klamt, A.; Jonas, V.; Burger, T.; Lohrenz, J. C. W. *J. Phys. Chem. A* **1998**, *102*, 5074-5085.

S21 Eckert, F.; Klamt, A. *AIChE J.* **2002**, *48*, 369-385.

S22 Eckert, F.; Klamt, A. COSMOtherm, Version C2.1, Release 01.10; COSMOlogic GmbH & Co KG, Leverkusen, Germany, **2009**.

S23 Kastenholz, M. A.; Hünenberger, H. *J. Chem. Phys*. **2006**, *124*, 224501

S24 Kelly, C. P.; Cramer, C. J.; Truhlar, D. G. *J. Phys. Chem. B* **2007**, *2*, 408-422

S25 Khobragade, D. A.; Mahamulkar, S. G.; Pospíšil, L.; Císařovμ, I.; Rulíšek, L.; Jahn, U. *Chem. Eur. J.* **2012**, *18*, 12267-12277

S26 Ben-Naim, A. Solvation Thermodynamics; Plenum: New York, **1987**.

S27 Meng, S. L.; Zhang, C.; Ye, C.; Li, J. H.; Zhou, S.; Zhu, L.; Li, X. B.; Tung, C. H.; Wu, L. Z. *Energy Environ. Sci.* **2023**, *16*, 1590–1596.

S28 Weatherburn, M. W. *Anal. Chem.* **1967**, *39*, 971−974.

S29 Schnidrig, S.; Bachmann, C.; Müller, P.; Weder, N.; Spingler, B.; Joliat-Wick, E.; Mosberger, M.; Windisch, J.; Alberto, R.; Probst, B. *ChemSusChem* **2017**, *10*, 4570-4580.

S30 Tong, L.; Kopecky, A.; Zong, R.; Gagnon, K. J.; Ahlquist, M. S. G.; Thummel, R. P. *Inorg. Chem*. **2015**, *54*, 7873-7884.

S31 Tong, L.; Zong, R.; Thummel, R. P. *J. Am. Chem. Soc.* **2014**, *136*, 4881-4884.

S32 Rountree, E. S.; McCarthy, B. D.; Eisenhart, T. T.; Dempsey, J. L. *Inorg. Chem*. **2014**, *53*, 9983-10002.

S33 Artero, V.; Saveant, J.-M. *Energy Environ. Sci.* **2014**, *7*, 3808-3814.

S34 Lucarini, F.; Fize, J.; Morozan, A.; Marazzi, M.; Natali, M.; Pastore, M.; Artero, V.; Ruggi, A. *Sustainable Energy Fuels* **2020**, *4*, 589-599.

S35 Pitre, S. P.; McTiernan, C. D.; Vine, W.; DiPucchio, R.; Grenier, M.; Scaiano, J. C. *Sci. Rep.* **2015**, *5*, 16397.

S36 Evans, D. F. *J. Chem. Soc*. **1959**, 2003-2005.

S37 Grant, D. H. *J. Chem. Ed*. **1995**, *72*, 39-40.
